# Improving the Safety and Quality of Care for Children and Young People With Intellectual and Developmental Disability. The Motivated for Change Programme in a Paediatric Emergency Department Setting. A Mixed Methods Study

**DOI:** 10.1111/hex.70776

**Published:** 2026-08-02

**Authors:** Natalie Ong, Sarah Ghasem Zadeh, Jerzy Zieba, Karl Pobre, Gail Tomsic, Jacqueline Milne, Shefali Jani, Natalie Silove

**Affiliations:** ^1^ Sydney Medical School, Faculty of Medicine and Health University of Sydney Camperdown New South Wales Australia; ^2^ Child Development Unit The Children's Hospital at Westmead Sydney New South Wales Australia; ^3^ Brain and Mind Research Centre University of Sydney Sydney New South Wales Australia; ^4^ Collegium Medicum, Faculty of Health Sciences and Psychology University of Rzeszów Rzeszów Poland; ^5^ Department of Paediatric Emergency The Children's Hospital at Westmead Sydney New South Wales Australia

**Keywords:** children and young people, continuing professional development, developmental disability, intellectual disability, quality improvement

## Abstract

**Background:**

Children and young people with intellectual and developmental disability have long been known to suffer from inequities in accessing safe and quality health care. These challenges are most felt within the emergency department due to its busy, crowded, and overstimulating environments with staff that are not trained to provide reasonably adjusted care.

**Aims:**

The study is an evaluation using qualitative and quantitative methods of a continuing education and quality improvement programme designed to increase the knowledge, skills, and confidence of healthcare staff in providing reasonable adjustments to children and young people with intellectual disability in hospitals.

**Methods:**

This study evaluated the Motivated for Change programme, which uses Behavioural change, Adult Learning, and Quality Improvement strategies to drive practice change and service improvement. A hundred and thirty‐one and 89 staff participated in a pre‐ and post‐intervention survey. Interviews were conducted with parents of children and young people with intellectual and developmental disability attending the emergency department. Observational studies were also conducted in the pre‐ and post‐intervention phase. These were transcribed, categorised, and coded with themes derived. Consensus was reached through meetings with the research team.

**Results:**

The Motivated for Change programme has demonstrated significant increases in staff knowledge, skills, and experience in providing safe and quality care for these children and young people. Evidence of change is reflected in the parent and staff interview and observational study themes. In addition, staff were further motivated to undertake quality improvement projects, develop an in‐house training programme to provide ongoing training for new staff, and make further improvements in the department.

**Conclusions:**

The Motivated for Change programme has demonstrated its efficacy in enabling changes in the practice of reasonable adjustments and the emergence of local champions for sustained efforts for ongoing improvements in safety and quality of care.

**Patient and Public Involvement:**

The development of the research question and outcome measures was informed by data from previous research publications involving parents and staff. We sought input from a parent advisory committee in the design of the study. Parents were not involved in the recruitment and conduct of the study. Results were presented to the parent advisory at the end of the study.

## 1 Background

People with intellectual or developmental (ID/DD) disability are known to be high users of the emergency department (ED) compared to the general population. They are twice as likely compared to the general population to present to the ED and seven times more likely to represent to the ED [1]. Despite their frequent interactions with the healthcare system, the complex needs of individuals with ID/DD are often inadequately addressed in the ED due to time constraints, resource limitations, and systemic inefficiencies [2]. They are also more likely to suffer patient safety events [3, 4, 5] due to a discomfort of staff to identify patients with suspected ID/DD, challenges in developing a system to flag patients with ID/DD in the ED, the need for educational resources to help adapt their communications and general approach to patients with ID/DD, lack of embedded tools within the ED care pathway to assist in providing discharge instructions, crowded environments and resource limitations of the departmental setting [5]. The data is similar for children and young people with intellectual and developmental disability (CYPIDD), being 1.8 times higher than the general population to present to ED and costing 2 times more per capita in hospital expenditure [6].

From the publication of the Mencap review in the United Kingdom to the current Royal Commission into the Abuse, Violence, Neglect and Exploitation of persons with ID in Australia, there has been an urgent call for the training of hospital staff to improve safety and quality health care for patients with ID/DD as part of a whole of health strategy to improve outcomes for this population [7, 8, 9, 10, 11, 12]. In addition, being knowledgeable and skilled in communicating, caring, and managing behaviours of CYPIDD are skills that have been found lacking in many studies, raising the need for widespread and effective training in the healthcare workforce [9, 13, 14, 15, 16, 17].

Educational and change behaviour experts suggest that for learning to transform into behaviour, it needs to be interactive, teach to task, involve facilitated group discussions, apply to the local context and influence intrinsic motivation for sustainability and diffusion [18, 19]. Utilising the COM B Framework [20], along with principles from adult learning theory, change psychology and organisational change [18, 21, 22], a pilot study utilising motivational interview (MI), flipped classroom and process mapping was trialled in a sleep study team, Kids Research and Medical Imaging at the Sydney Children's Hospitals Network (SCHN) [23, 24] This paper highlighted the importance of using personal experiences, group norming, facilitated discussions and application in practice in quality improvement cycles to transform learning into practice changes in an Action Research paradigm. These novel techniques have been developed into a programme titled ‘Motivated for Change’ programme and is currently being trialled across the SCHN. This study will focus on the implementation of this programme in the Paediatric Emergency Department at The Children's Hospital at Westmead (CHW ED). The CHW ED is a tertiary paediatric ED which serves upwards of 65000 children and young people per year.

### 1.1 Session Outline

This Continuing Professional Development – Quality Improvement (CPD‐QI) programme integrates MI techniques, flipped classroom methodology and process mapping strategies along simulation‐based education to embed knowledge into practice and promote staff agency [23]. SMART goals were agreed upon at the end of the second session, and an integrated process map was developed at the end of all second sessions. See Table 1.

**Table 1:** Session Structure and Content.

Session 1	Session 2	Session 3
Pre‐session videos on ‘What is intellectual disability’, Family perspectives and ‘What are reasonable adjustments?’	**Recap**	Follow up
Introductions and session outline	Introductions and session outline	Introduction of follow‐up
Understanding developmental disability in the healthcare context	Simulation scenario (run by simulation coordinator)	Elicitation of session experience, and if any adaptations were discussed and implemented
Sharing experiences of caring for a patient with DD and challenging behaviours – MI	Process Mapping (PM) – List stages of the journey	Review of the process map and if SMART goals have been achieved
Causes of behavioural problems in DD	List touchpoints and feelings – MI	Enablers and barriers to practice and process adaptations
Learning from others/literature about reasonable adjustments – MI		
**What can be done in one's particular clinical environment? MI**	List changes and necessary adjustments	Review of goals
	Set goals (SMART) – MI	
Follow date was arranged	Evaluation and readiness to act – MI	Future plans
Session close	Session close	Session close

### 1.2 Aims

The primary aim of the study is to evaluate the efficacy of the CPD‐QI programme in enhancing self‐reported and observed improvements in knowledge, skills, attitudes, and behaviours in clinical practice of staff who care for CYPIDD.

The secondary aim of the study seeks to evaluate the experiences of parents of CYPIDD who have presented to the ED for care and examine the possible effects of the programme on their experiences of care. Additionally, the study examines whether the length of tenure or prior experience in health care correlates with levels of knowledge, confidence, and skills in caring for this population.

## 2 Materials and Methods

The research team conducted an action research–informed mixed methods pre–post cohort study. Action Research is an iterative process of reflection, action, and evaluation for improving educational or clinical practice [25, 26]. It involves gathering evidence in a reflexive, participative, and collaborative way and enables problem‐solving and practice changes. The approach is situational and context‐based, with participants interpreting situations, thus leading to knowledge creation or problem‐solving through action and practice improvements. Action plans are created, implemented, revised, and then re‐implemented, supporting ongoing reflection and revision [27]. The effects of the programme on staff's understanding, knowledge, skills, and attitudes were studied by comparing before and after programme delivery to staff at CHW, including after 6–8 months post‐training of participating staff. This consisted of a series of interviews, observational studies and self‐rated surveys examining the knowledge, skills and confidence of staff, parent and staff interviews and observational studies in the pre (November 2022–January 2023) and post (November 2023–January 2024) training time periods. Please see Supporting Information S1: Data 1 – (a) pre‐ and post‐intervention surveys.

The development of the research question and outcome measures was informed by data from previous research publications involving parents and staff [28, 29]. We sought input from a parent advisory committee in the design of the study. Parents were not involved in the recruitment and conduct of the study. Results were presented to the parent advisory at the end of the study.

All ED staff, including nurses, doctors, and allied health staff across all seniority levels, were recruited via an email flyer containing information about the programme and research project, which was emailed to every staff member in the department. Participation was voluntary, initiated through the completion of the pre‐session survey adapted from a validated survey previously used to assess staff knowledge, attitudes and confidence caring for patients with ID and mental health issues [30].

Parent and staff interviews and staff observations were conducted at the ED by the research team in the same pre‐ and post‐training periods.

Posters were put up on the walls of the department informing parents that the research team were conducting observational studies and those who did not want to be observed could approach the team and inform them of the opt‐out. Parents who consented to be interviewed had scanned a QR code from a wall‐mounted poster providing details to be contacted. Observations were carried out by a research officer, a postgraduate student, a nurse, and a paediatric trainee, using an observation schedule with written field notes for data triangulation. Observational methods were chosen for their effectiveness in studying organisational dynamics and uncovering behaviours or routines that participants may not be consciously aware of [31, 32, 33].

The research team engaged with staff and notified them about the observation period prior to commencing. Observed staff had the option to withdraw from being observed. Sessions ran periodically for 1–2 h during the day, any day of the week. Posters were placed in the ED to inform parents that research observations were underway. Parents who did not wish to participate in the observation were encouraged to inform staff or a member of the research team. Saturation was achieved with observations, but due to difficulties recruiting parents for the interview, coding was completed as recruited.

All interviews were transcribed via Teams transcription, and the resulting interview scripts and observer field notes were used in data analysis.

Training sessions and staff interviews were delivered to ED staff between March and June 2023, during which pre‐training staff surveys were collected prior to each session. Post‐training staff surveys, as well as parent and staff interviews and observations, were conducted at CHW between November 2023 and January 2024. Ethics approval was obtained through the SCHN HREC 2022_ETH01993.

**Figure 1:**
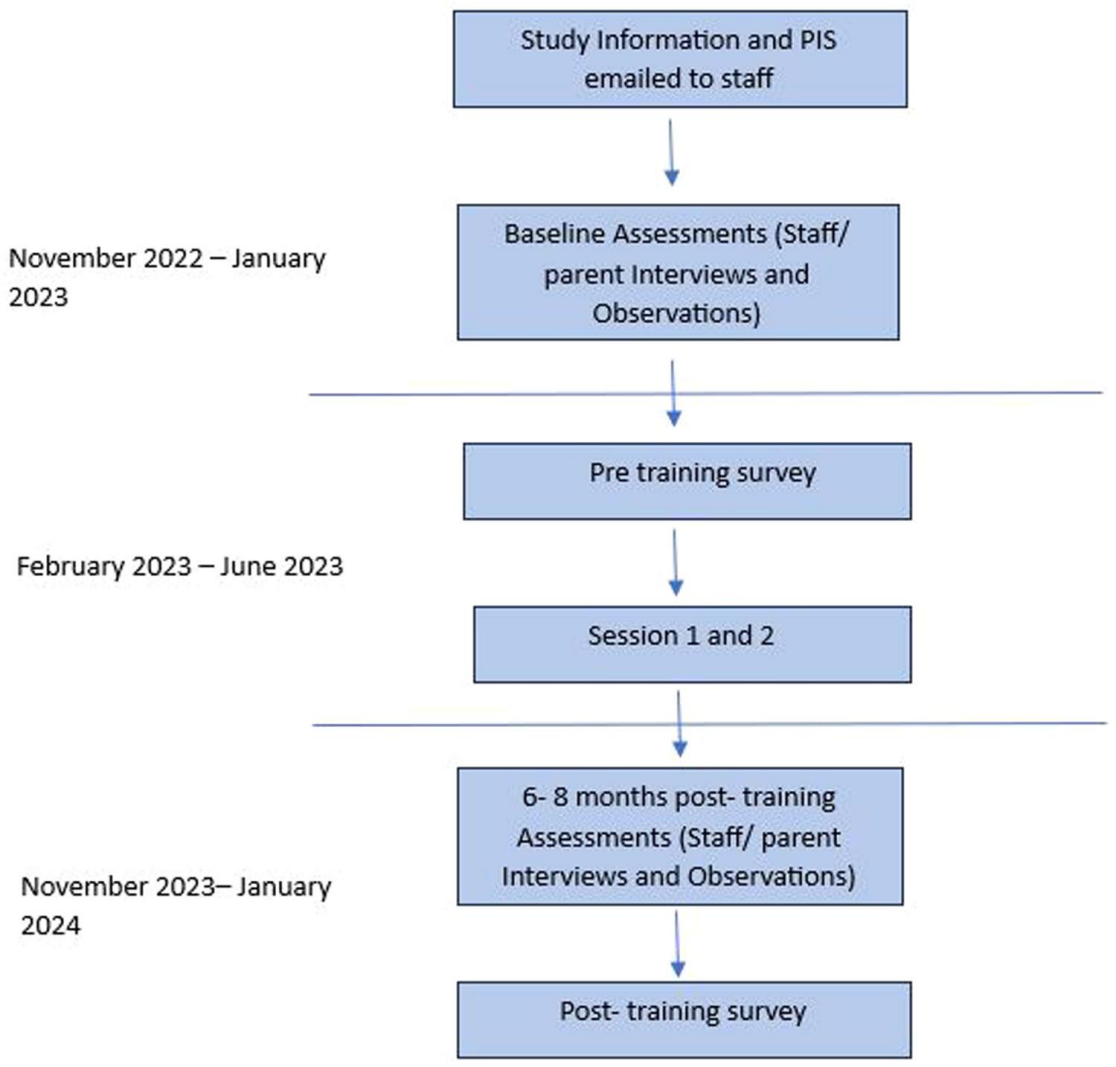
Study timeline.

The flow of the study is shown in Figure 1 below.

## 3 Results

This study used interviews and observations to assess the impact of training on healthcare workers' knowledge and skills in working with children and young people with disabilities, and if there were changes noted in post‐session regarding staff behaviour and the patient experience. There were 4 pre‐ and 4 post‐parent interviews, 6 pre‐ and 7 post‐staff interviews, and 11 pre‐ and 13 post‐session observations conducted during the study period. In addition, pre‐ and post‐session surveys were also completed by participating staff. A total of 131 pre‐surveys and 89 post‐surveys were collected. In addition, a total of 39 paired surveys from nurses (*n* = 21), doctors (*n* = 16), and other healthcare workers (*n* = 2) were analysed for individual change. Statistical analysis was conducted using IBM SPSS Statistics 29, applying nonparametric tests due to the ordinal nature of the data. The Framework Method was used for data analysis, where the codes were organised into a framework, and additional codes were grouped into existing or new categories. Thematic analysis was derived from the codes and categories. This method is often used in qualitative evaluations [34]. A bracketing exercise was conducted where researchers temporarily set aside their own preconceived notions, biases, and assumptions to better understand participants' experiences and perspectives [35].

### 3.1 Qualitative

#### 3.1.1 Improvements Noted by Parents

Parents were reporting both on interview and during observations that they had noticed significant changes in the way staff interacted with the child, worked in partnership with them and included the Child Life therapist. Staff were noted to use the top 5 (electronic flagging system for reasonable adjustments)/hospital passport in guiding communication and care coordination. This helped with timeliness and streamlining of care, information transfer, better modification of the environment and better child engagement.

And then she got the Top 5 done, and then she (Child Life Therapist) consulted with the medical team and staff. … I think it makes a difference because that never happened to me before and I think that is a brilliant thing to have in place. It was a more positive experience, and I have ever experienced before.

Parent 1

#### 3.1.2 Improvements Noticed by Staff

Staff reported improvements in their agency and confidence in providing care. They alluded to previously being very anxious and uncertain about what to do. However, with greater knowledge, skills and confidence, they have noticed that this has resulted in greater comfort with the child. They have also noticed that their adjustments were beginning to have a flow‐on impact on other departments. Staff were more aware of asking about the child's need for adjusted care rather than solely focusing on medical complexity. Staff were surprised at the positive outcomes that they had experienced and attributed to the training programme.

So I guess after the train the trainer [program] … was good insight and for all that was just to reflect on those difficulties and to have a better approach of these patients. So definitely [it] was very eye‐opening. It is just sometimes we know what to do but is just putting them altogether in a more structured way … definitely was an eye opener and definitely helps with the anxiety feeling before seeing patients.

Staff 2

### 3.2 Increased Staff Enthusiasm and Optimism

There were reports of staff feeling that the programme had made a difference to the way they provided clinical care. There was a belief that one day this would be standard practice and had trust in the leadership to champion this on an ongoing basis. Staff showed eagerness to be part of future projects in resource development.

…approaching them a bit more differently definitely helped and just made that whole interaction very smooth and actually [parents] thanked me a lot after I finished the consultation, which was very surprising to me given the fact that everyone's saying [they were] a very difficult family and they actually warned me before entering the room. So I guess I must have done something really good with that training, yeah.

Staff 2

#### 3.2.1 Types of Reasonable Adjustments Observed

The types of reasonable adjustments observed were primarily around adjustments centred on the child. Both staff and parents report on the presence of Child Life Therapists more in the postintervention phase than in the preintervention phase. Low sensory measures were undertaken with staff being a lot more opportunistic. Staff demonstrated a better response to escalation prevention, for example, considering early the use of anti‐anxiolytics and parent enquiry before escalation. Care was provided in a more adapted and timely way within the dynamics of the team setting. Modelling desirable behaviours when engaging with the child benefited trainees who were observing more senior clinicians demonstrating the potential for these skills to be transferred to more inexperienced staff members (outside of formal training).

And when I did the same, it was amazing how much they absorb just from the teaching that one teaching session they have already, you know implementing what they learned from that session.

Staff 6

#### 3.2.2 Tile Noted to be Used and Championing Its Use

The Top 5/All about me tile was noted to be used during observations and staff reports. This is an electronic flagging system which identifies a child in need of reasonable adjustments. It provides advance notice to staff at the triage level for them to be able to respond with appropriate, reasonable adjustments. Staff were also surveying the waiting area and approaching parents to complete the Top 5/All About Me tile if there was a child identified to have a neurodevelopmental condition. This tile was also reported to be beneficial to other departments in improving the transfer of information on the need for reasonable adjustments when the child is admitted to the ward or for tests or procedures external to the ED. There was potential for other applications of the tile in research. It was felt that the tile provided better continuity of care, and its flexibility in the capacity for revision with more effective strategies was highlighted. The tile also serves as a large factor for staff to ask parents key questions about the need for their child for reasonable adjustments. However, it was also noted that the use of the tile was inconsistent depending on whether the staff received said training or not.

We've had a few changes in the department where there's an [electronic] tile now which pops up and once we put, you know, if we flag the child as having intellectual disability and it there's a page that pops up which gives you the top five things that the child likes.

Staff 1

#### 3.2.3 Environment Is Variable and Unpredictable

There was an acknowledgement that whilst significant improvements were noted by parents and staff about departmental changes, there were some things that were more challenging to adapt to. The lack of control over the noise and crowded settings of the Department was seen to be an ongoing challenge. The variability and unpredictability of the ED will always be there, and the staff were cognisant that, in spite of these challenges, there was an ever‐increasing need to still be able to provide safe and good care.

This kid's crying, screaming. The nurses station is busy. It's it's loud, there's buzzing noises going off and it was a loud environment.

Parent 2

There may not be a single room to put them in somewhere, and even giving them a slightly high category their wait time may be extended…. it's sort of nothing you can do about it.

Staff 5

For more qualitative data, see Supporting Information S1: Data 2.

### 3.3 Quantitative

The Wilcoxon test revealed statistically significant improvements in subjective indicators of knowledge, skills, and experience across all domains following the training. Notable improvements included working with children with ID/DD (*p* < 0.001), physical disabilities (*p* = 0.005), challenging behaviours (*p* = 0.002), and communication difficulties (*p* < 0.001). Additionally, there was a significant increase in knowledge and skills related to strategies for supporting communication and managing challenging behaviours (*p* < 0.001).

Further analysis showed that both doctors and nurses demonstrated significant improvements, with nurses showing stronger enhancements in 13 out of 14 variables. For doctors, significant improvements were observed in knowledge and skills concerning IDs (*p* = 0.038), communication difficulties (*p* = 0.002), and strategies for managing challenging behaviours (*p* = 0.020). Among nurses, significant improvements were found in knowledge related to communication (*p* = 0.002) and challenging behaviours (*p* < 0.001).

Finally, correlations between work experience, frequency of contact with disabled patients, and self‐perceived knowledge were examined. While no significant correlations were found for doctors, among nurses, pre‐training contact frequency was positively correlated with knowledge and skills, but post‐training, this correlation diminished. This suggests a flattening effect in self‐assessed competence post‐training. For more information, please see Supporting Information S1: Data 1 – (b) Table of Results.

## 4 Discussion and Conclusion

This study demonstrates the efficacy of a CPD‐QI programme that utilises a co‐design approach, integrating strategies in adult learning, behaviour change and quality improvement to enhance uptake and implementation of reasonable adjustments in the ED setting. The combination of Motivational Interviewing, Flipped Classroom, Simulation‐based education and Process Mapping enabled staff to operationalise their knowledge and develop agency to implement these adjustments effectively.

Significant improvements were observed in the post‐session staff surveys across the stated competencies, and these gains were maintained in individual analyses and subgroup analyses for doctors and nurses. Interestingly, in the post self‐assessment for nurses, the flattening out effect suggests that the programme had a ‘levelling out’ effect, indicating the programme's efficacy in enhancing self‐perceived knowledge and skills of the less experienced to the level of those who were more experienced.

Qualitatively, the weight of the analysis was based on the triangulation of observational findings with parent and staff data, which were exploratory in nature. Both qualitative and quantitative analyses served to demonstrate evidence of change, not only at the clinician level but also at the departmental level and experienced by both parents and staff.

Current literature exploring de‐escalation programmes for children with autism focuses on de‐escalation skillsets, for example, addressing de‐escalation using verbal strategies and through simulation education [36, 37]. Other de‐escalation programmes have a broader remit and include escalation from patients with mental health, conduct issues or other causes, not unique to the patient with ID/DD [19]. The MFC programme endows staff with the capability of adaptive responses in embedding reasonable adjustments which are relevant to the local context [19, 24]. It also has a preventative focus which includes addressing safety and quality of care in addition to early identification of distress and the appropriate application of de‐escalation strategies [24].

This iteration of the programme has resulted in the formation of an in‐house team dedicated to sustaining the programme over the long term. The programme is embedded within the department's medical and nursing teaching schedules on a regular periodic basis. We believe that the key sustainability factor is the development of local champions within the department who take ownership of the programme's implementation and the resultant QI projects that stem from it. Further research is required to examine the ‘local champion’ phenomenon in the next iteration of the programme, which is currently in progress, to understand the factors that enhance the presence of champions. Transferability of the programme will rest on the ability to develop a core training team to run ‘Train the trainer’ initiatives/programmes for transferability to other health contexts.

The programme also facilitated the creation of process maps that provided clarity and direction for areas requiring improvement, leading to the development of several spin‐off QI projects. See Supporting Information S3: Data 3 and Supporting Information S4: Data 4. As found in previous studies, the Motivated for Change programme is a model that demonstrates how one can facilitate change through a combination of strategies that target staff education, motivation, practice and service improvement in a cohesive way that leads to change in clinical practice. When the leadership of a department and parent involvement are included at the outset, this reinforces the transmission of knowledge through the layers from clinical practice to departmental and leadership levels.

**Figure 2:**
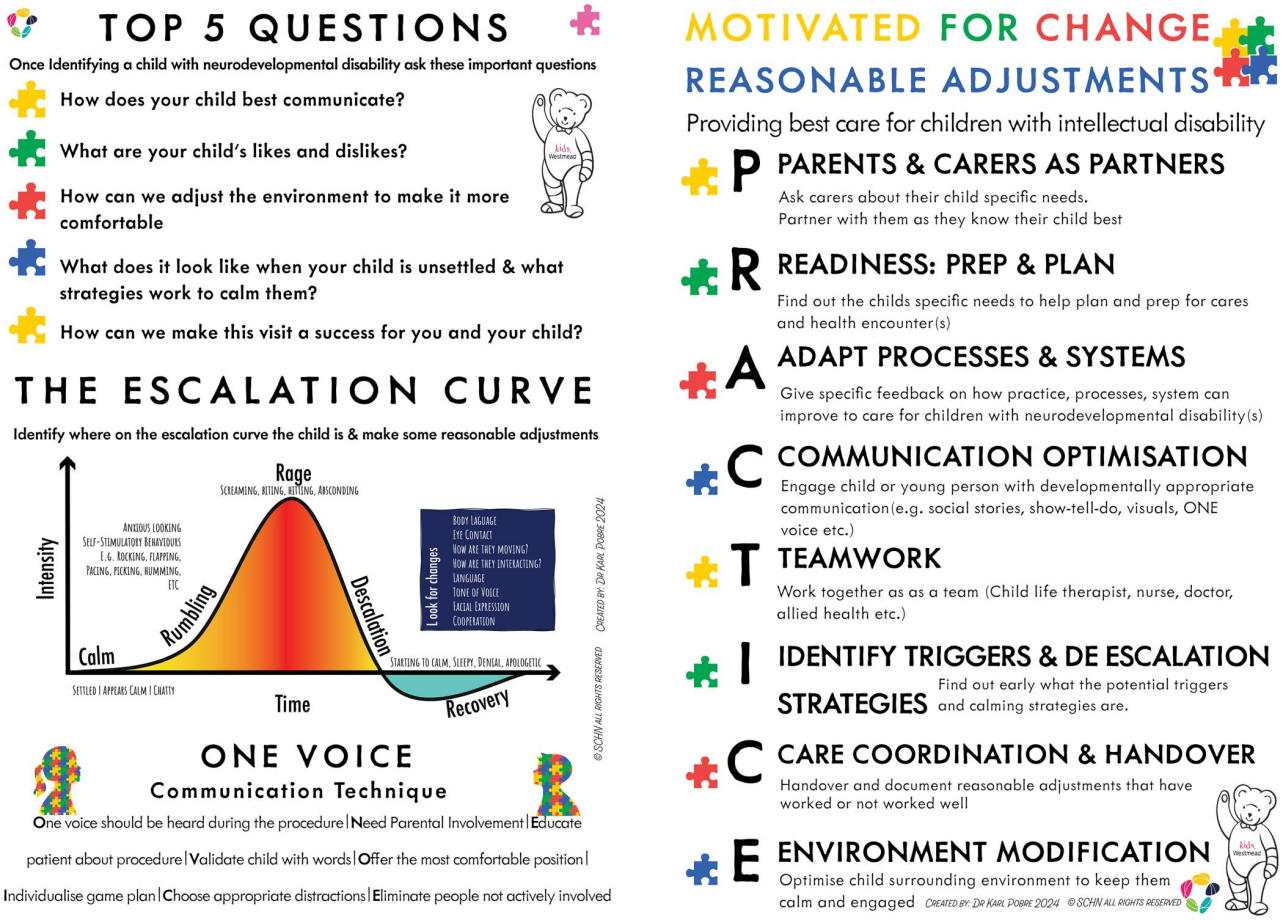
Staff lanyard on reasonable adjustments.

**Figure 3:**
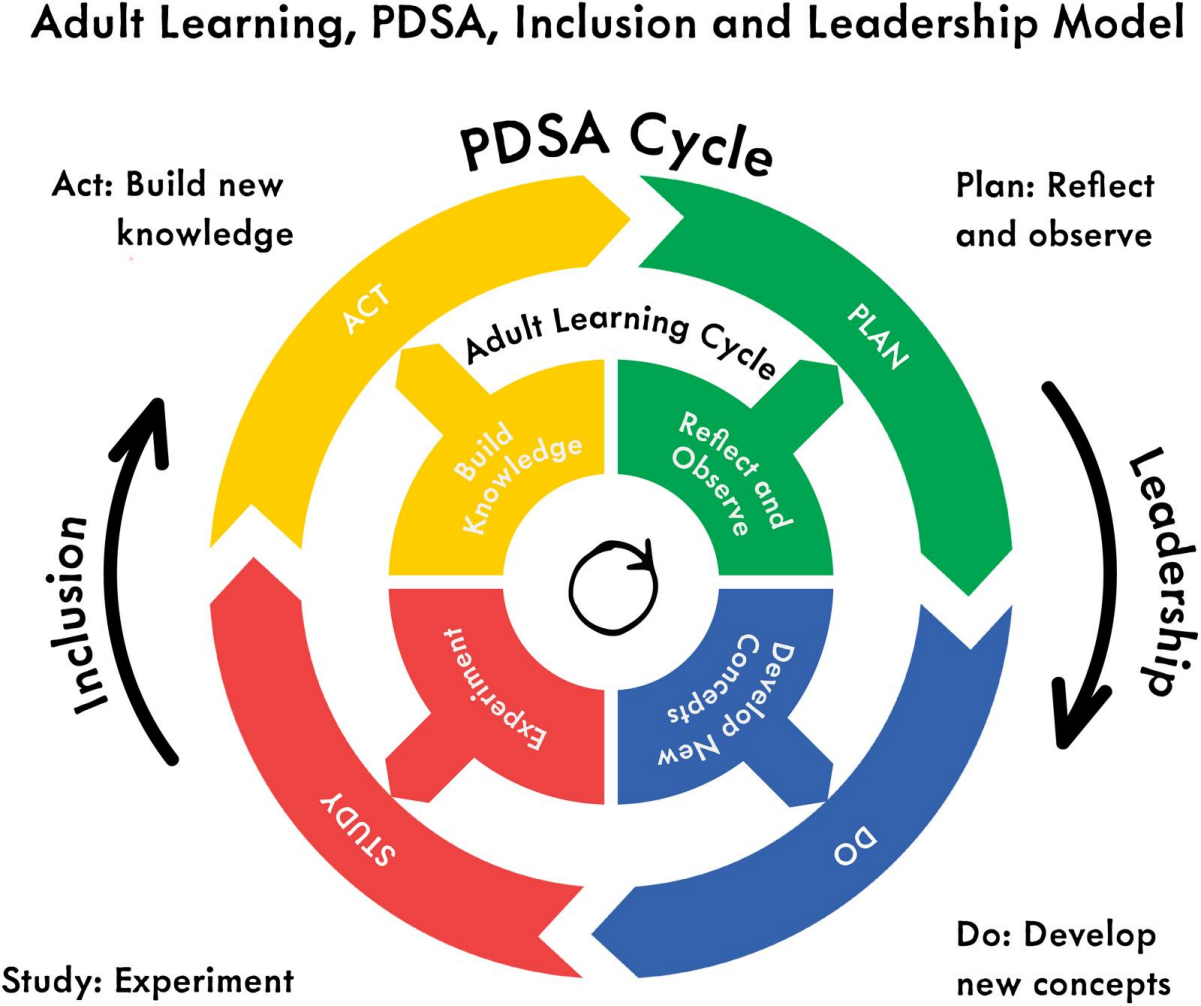
Hybrid adult learning – CPD model. Incorporating inclusion and leadership for greater change.

See Figure 2 (Staff lanyard on reasonable adjustments [developed by staff]) and Figure 3 (The hybrid adult learning – CPD model).

## 5 Limitations

Limitations comprise those inherent to the study design and to data availability. This programme was conducted using an Action Research paradigm, which measures change through pre‐ and post‐data analysis, eliminating the need for control groups. While the study provided evidence of improved patient/parent experience, there were no quantitative measures of patient outcomes. To further strengthen the evaluation of the programme's impact, future programme iterations should incorporate hospital data such as incident management reports, code blacks, and parent complaints. These proposed indicators represent a move towards more objective measures of safety and quality of care. We also acknowledge the relatively low number of interviews with parents and staff. It would have been desirable to recruit more parents and staff; however, the busy and stressful nature of the ED poses great challenges in participant recruitment, as other competing priorities exist. Whilst the interviews were more exploratory in nature and triangulated with the observational data well, more efficient ways to gain qualitative feedback need to be further explored to refine future research activities and inform programme improvements.

### 5.1 Future Directions

This programme has significant potential for translation to other acute care departments, where many of the challenges pertaining to the care of this population are experienced. In increasing reach, the development of an online platform for blended learning sessions could be useful for other regional and rural healthcare services. Given the busy nature of acute care departments, there is scope to increase the flexibility of sessions to be condensed further for shorter sessions by pairing with pre‐session videos and reflective exercises. Finally, the format of these sessions can be applied across topics and healthcare areas where there is a need for change and improvement.

## Author Contributions

**Natalie Ong:** conceptualisation, investigation, funding acquisition, writing – original draft, methodology, writing – review and editing, formal analysis. **Sarah Ghasem Zadeh:** writing – original draft, writing – review and editing, formal analysis. **Jerzy Zieba:** validation, writing – review and editing, formal analysis. **Karl Pobre:** writing – original draft, writing – review and editing, formal analysis. **Gail Tomsic:** conceptualisation, methodology, project administration, writing – review and editing. **Jacqueline Milne:** writing – original draft, writing – review and editing, formal analysis. **Shefali Jani:** conceptualisation, methodology, writing – review and editing. **Natalie Silove:** conceptualisation, writing – review and editing, supervision.

## Ethics Statement

Ethics approval was granted from the Sydney Children's Hospitals Network Human Research Ethics Committee (SCHN HREC 2022_ETH01993).

## Conflicts of Interest

The authors declare no conflicts of interest.

## Supporting information


Supporting File 1

Supporting File 2

Supporting File 3

Supporting File 4

## Data Availability

The datasets used and/or analysed during the current study are available from the corresponding author on reasonable request.
