# Supplementary material for: Improving the Safety and Quality of Care for Children and Young People With Intellectual and Developmental Disability. The Motivated for Change Programme in a Paediatric Emergency Department Setting. A Mixed Methods Study
Source: Health Expect. 2026 Aug 2;29(4):e70776. doi: 10.1111/hex.70776 (PMC13429100; doi:10.1111/hex.70776)
Supplement: Supplementary file 1 — Supporting File 1 [file HEX-29-e70776-s001.docx]

Pre-Session Questionnaire for Motivated For Change Program

| Questionnaire Name |  |
| --- | --- |
| Standard |  |
| Patient Oriented |  |
| Questionnaire Instruction |  |
| Creator Name |  |
| Creator Group |  |

 **Participant Information: Improving the care of children with intellectual disability in hospital (Ethics number: 2020_ETH02240)**

Dear staff,

We are conducting this research survey to better understand your views on your knowledge and skills caring for children and young people with intellectual disability in hospital.

Your responses will assist us in better understanding what we need to change. Your responses will be deidentified and analysed anonymously and not affect your standing in hospital. Any identifying data collected in this survey will be removed and it will be separately stored in a password protected drive within the SCHN, including the details of participants who have consented to participate in an interview.

By completing this survey, you are consenting to participate in this research project. If you have any questions about the project, please email us at natalie.ong@health.nsw.gov.au


[Staff Participant Information Sheet](https://nswhealth-my.sharepoint.com/:b:/g/personal/gail_tomsic_health_nsw_gov_au/EVPQoaLxb11BlEs3gWjnLSMBa21xk4ziq2ob_LTnnR2N1A?e=xR3Mmu)

Thank you.

| 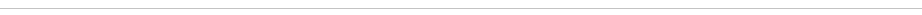 | |
| --- | --- |
| ***1.** | Which hospital(s) are you currently employed by? [Question ID: 78504] |
|  | ●  ○  The Children's Hospital at Westmead ○  Sydney Children's Hospital ○  Both |
| 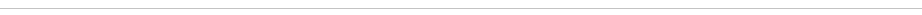 | |
| **2.** | Please enter your stafflink number: [Question ID: 82078] |
|  | \|  \| \| --- \| |
| 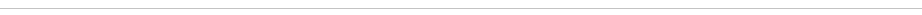 | |
| ***3.** | What is your professional background? [Question ID: 78505] |
|  | ●  ○  Nursing ○  Medical ○  Allied Health ○  Other |
| 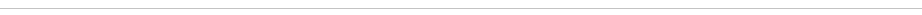 | |
| ***4.** | What is your current position? [Question ID: 78506] |
|  | \|  \| \| --- \| |
|  | **Comments**: |
| 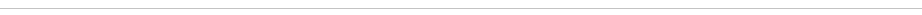 | |
| ***5.** | . Which environment best describes your workplace? (Please identify all areas relevant) [Question ID: 78507] |
|  | □ Triage □ Resus area □ Cubes □ Short Stay □ Observation □ Other |
| 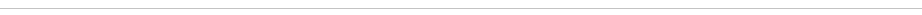 | |
| ***6.** | How many years have you been working in healthcare? [Question ID: 78508] |
|  | ●  ○  0-2 years ○  3-5 years ○  6-10 years ○  11-14 years ○  +15 years |
| 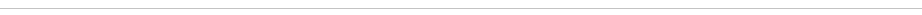 | |
| ***7.** | On average, how much professional contact do you have with people who have an  intellectual disability? [Question ID: 78509] |
|  | ●  ○  None ○  Daily ○  weekly ○  monthly ○  yearly |
| 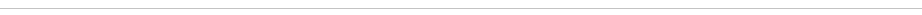 | |
| ***8.** | On average, how much non-professional contact do you have with people with intellectual disabilities? [Question ID: 78510] |
|  | ●  ○  None ○  daily ○  weekly ○  monthly ○  yearly |
| 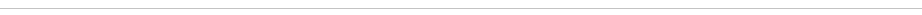 | |
| ***9.** | Please rate: Overall how successful do you feel have these interactions with people with intellectual disability been (professional and non- professional)? Where 1 is very poor up to 10 Very successful (numeric field with 10 decimal point.) [Question ID: 78511] |
|  | \|  \| \| --- \|   *(1)* |
|  | **Comments**: |
| 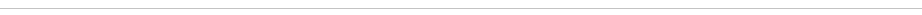 | |
| ***10.** | I feel I have the necessary knowledge, training and skills to manage successfully with people with an intellectual disability in my healthcare environment. [Question ID: 78512] |
|  | ●  ○  Strongly disagree ○  Disagree ○  Neutral ○  Agree ○  Strongly agree |
| 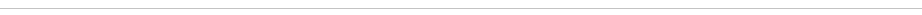 | |
| ***11.** | I feel I have the necessary knowledge, training and skills to manage successfully people with a range of communication skills and difficult behaviours in my healthcare environment. [Question ID: 78514] |
|  | ●  ○  strongly disagree ○  diagree ○  neutral ○  agree ○  strongly agree |
| 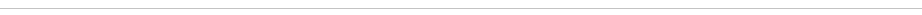 | |
| ***12.** | I believe that treating people with intellectual disability is a part of my role/s as a health professional. [Question ID: 78515] |
|  | ●  ○  Strongly disagree ○  Disagree ○  Neutral ○  agree ○  strongly agree |
| 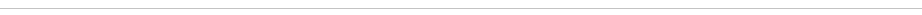 | |
| ***13.** | I believe that treating people with intellectual disability is an expectation from my colleagues and my professional body. [Question ID: 78516] |
|  | ●  ○  strongly disagree ○  disagree ○  neutral ○  agree ○  strongly agree |
| 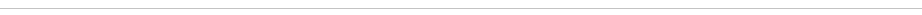 | |
| ***14.** | The physical and mental health needs of people with intellectual disability can be adequately met in a mainstream health service [Question ID: 78519] |
|  | ●  ○  strongly disagree ○  disagree ○  neutral ○  agree ○  strongly agree |
| 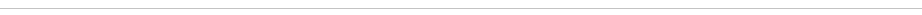 | |
| ***15.** | My knowledge, training and skills are easily implemented in my healthcare practice and work environment when I contact patients with intellectual disability. [Question ID: 78520] |
|  | ●  ○  strongly disagree ○  disagree ○  neutral ○  agree ○  strongly agree |
| 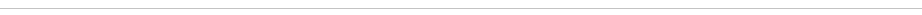 | |
| ***16.** | The physical and mental health of people with intellectual disability is no better or worse than people without intellectual disability. [Question ID: 78521] |
|  | ●  ○  strongly disagree ○  disagree ○  neutral ○  agree ○  strongly agree |
| 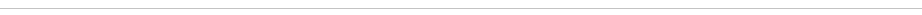 | |
| ***17.** | Even if it is hard to understand people with intellectual disability, I think it is still important to talk to them personally [Question ID: 78530] |
|  | ●  ○  Strongly disagree ○  disagree ○  neutral ○  agree ○  strongly agree |
| 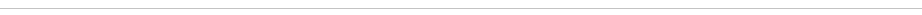 | |
| ***18.** | I would not be able to spend time and explain treatment plans to a person with intellectual disability as they did not understand [Question ID: 78535] |
|  | ●  ○  strongly disagree ○  disagree ○  neutral ○  agree ○  strongly agree |
| 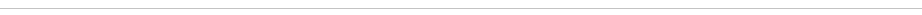 | |
| ***19.** | I would like to have information given to me about the patient with an intellectual disability who will use my health services during their health admission/treatment [Question ID: 78537] |
|  | ●  ○  strongly disagree ○  disagree ○  neutral ○  agree ○  strongly agree |
| 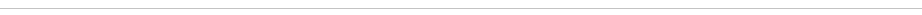 | |
| ***20.** | When working with a patient who has an intellectual disability, I make time to ensure that I am clear on what their health difficulty is, even if that means I run over schedule [Question ID: 78540] |
|  | ●  ○  strongly disagree ○  disagree ○  neutral ○  agree ○  strongly agree |
| 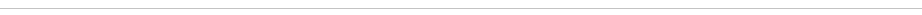 | |
| ***21.** | Carer/family members/relatives are a vital resource in providing information and support about the person with an intellectual disability. Even if a patient with an intellectual disability has good communication skills, I still find it more useful to talk to the carer first about their illness rather than the child your young person with intellectual disability. [Question ID: 78544] |
|  | ●  ○  strongly disagree ○  disagree ○  neutral ○  agree ○  strongly agree |
| 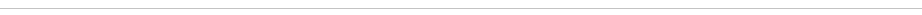 | |
| ***22.** | I always try and explore the health complaints of a patient who has an intellectual disability by communicating with the patient themselves, than solely asking their carer. [Question ID: 78547] |
|  | ●  ○  strongly disagree ○  disagree ○  neutral ○  agree ○  strongly agree |
| 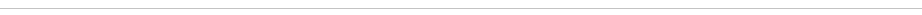 | |
| ***23.** | Please rate your knowledge, skills and experience working with people with development disability [Question ID: 78550] |
|  | \|  \| very low \| low \| neutral \| high \| very high \| \| --- \| --- \| --- \| --- \| --- \| --- \| \| Knowledge \| ○ \| ○ \| ○ \| ○ \| ○ \| \| Skills and Level of Experience \| ○ \| ○ \| ○ \| ○ \| ○ \| |
| 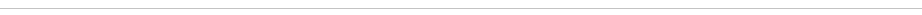 | |
| ***24.** | Please rate your knowledge, skills and experience in working with people with a physical disability [Question ID: 78554] |
|  | \|  \| very low \| low \| neutral \| high \| very high \| \| --- \| --- \| --- \| --- \| --- \| --- \| \| Knowledge \| ○ \| ○ \| ○ \| ○ \| ○ \| \| Skills and Level of Experience \| ○ \| ○ \| ○ \| ○ \| ○ \| |
| 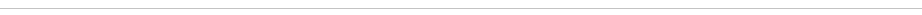 | |
| ***25.** | Please rate your knowledge, skills and experience in working with people with an intellectual disability and challenging behaviours [Question ID: 78556] |
|  | \|  \| very low \| low \| neutral \| high \| very high \| \| --- \| --- \| --- \| --- \| --- \| --- \| \| knowledge and skills \| ○ \| ○ \| ○ \| ○ \| ○ \| \| level of experience \| ○ \| ○ \| ○ \| ○ \| ○ \| |
| 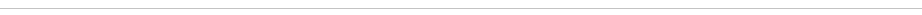 | |
| ***26.** | Please rate your knowledge, skills and experience in working with communication difficulties experienced by people with intellectual disabilities [Question ID: 78558] |
|  | \|  \| very low \| low \| neutral \| high \| very high \| \| --- \| --- \| --- \| --- \| --- \| --- \| \| knowledge and skills \| ○ \| ○ \| ○ \| ○ \| ○ \| \| level of experience \| ○ \| ○ \| ○ \| ○ \| ○ \| |
| 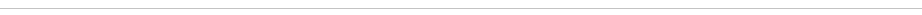 | |
| ***27.** | Please rate your knowledge, skills and experience in working with strategies used to support the communication of people with intellectual disabilities (e.g. Picture boards, signing, social stories, picture timetables) [Question ID: 78559] |
|  | \|  \| very low \| low \| neutral \| high \| very high \| \| --- \| --- \| --- \| --- \| --- \| --- \| \| Knowledge \| ○ \| ○ \| ○ \| ○ \| ○ \| \| Skills and Level of Experience \| ○ \| ○ \| ○ \| ○ \| ○ \| |
| 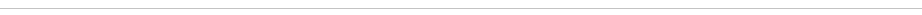 | |
| ***28.** | Please rate your knowledge, skills and experience in working with triggers and causes of challenging behavioural issues in people with intellectual disabilities [Question ID: 78560] |
|  | \|  \| very low \| low \| neutral \| high \| very high \| \| --- \| --- \| --- \| --- \| --- \| --- \| \| Knowledge \| ○ \| ○ \| ○ \| ○ \| ○ \| \| Skills and Level of Experience \| ○ \| ○ \| ○ \| ○ \| ○ \| |
| 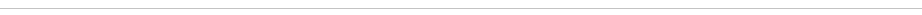 | |
| ***29.** | Please rate your knowledge, skills and experience in working with strategies to manage challenging behaviours in people with intellectual disabilities [Question ID: 78561] |
|  | \|  \| very low \| low \| neutral \| high \| very high \| \| --- \| --- \| --- \| --- \| --- \| --- \| \| Knowledge \| ○ \| ○ \| ○ \| ○ \| ○ \| \| Skills and Level of Experience \| ○ \| ○ \| ○ \| ○ \| ○ \| |
| 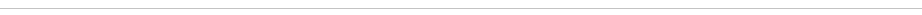 | |
| ***30.** | Please identify what you feel are contributing causes of difficulties experienced by patients and their families with an intellectual disability during their hospital admission and subsequent care. You may indicate more than one point. [Question ID: 78564] |
|  | □ communication □ unable to understand □ frustration from communication difficulties □ unrealistic expectations □ physical limitations □ emotional regulation □ sensory stimulation □ unwanted personal/physical contact with staff □ challenging behaviours □ ritualistic behaviour □ compulsive behaviour □ new environment □ time constraints of healthcare woprker □ adjustment to ward routine(s) □ environmental stimulation such as noise □ environmental stimulation such as busy ward areas □ none of the above □ Other |
|  | **Comments**: |
| 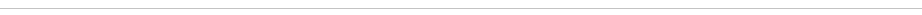 | |
| ***31.** | Are you aware of the various policies?      [Question ID: 78567] |
|  | \|  \| yes \| no \| \| --- \| --- \| --- \| \| NSW health Policy Directive, Disability-People with disabilities:responding to their needs during Hospitalisation \| ○ \| ○ \| \| Pharmacological strategies for children with developmental disability during investigations/procedures \| ○ \| ○ \| \| Developmental Disability Patients – Acute Management Plan Flowchart \| ○ \| ○ \| \| Non-restrictive Care for Mental Health Paediatric Inpatients with Co-morbid Intellectual Disability and/or Autism Spectrum Disorder \| ○ \| ○ \| |
| 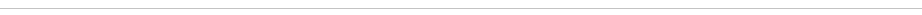 | |
| **32.** | If you have read the policies please indicate whether 1/2/3/4 (can pick more than one) [Question ID: 82104] |
|  | □ 1. NSW health Policy Directive, Disability-People with disabilities:Responding to their needs during Hospitalisation □ 2. Pharmacological strategies for children with developmental disability during investigations/procedures □ 3. Developmental Disability Patients – Acute Management Plan Flowchart □ 4. Non-restrictive Care for Mental Health Paediatric Inpatients with Co-morbid Intellectual Disability and/or Autism Spectrum Disorder |
| 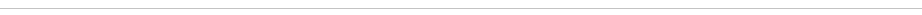 | |
| ***33.** | To the best of your knowledge, do you feel these policies have been implemented in your care setting? [Question ID: 78570] |
|  | ○ Yes ○ No |
| 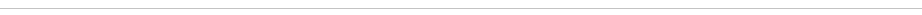 | |
| ***34.** | How much formal training in total have you received about working with people with intellectual disability? [Question ID: 78571] |
|  | ○  None   ○  1-3 hours   ○  3-6 hours   ○  1-4 days   ○  1-4 weeks   ○  more than 4 weeks |
| 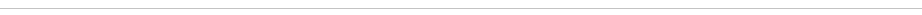 | |
| **35.** | Please list some examples of formal training you have had: [Question ID: 78574] |
|  | \|  \| \| --- \| |
| 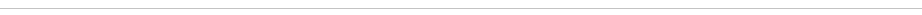 | |
| ***36.** | How much informal training have you received about working with people with intellectual disability? [Question ID: 78576] |
|  | ○  None   ○  1-3 hours   ○  3-6 hours   ○  1-4 days   ○  1-4 weeks   ○  More than 4 weeks |
| 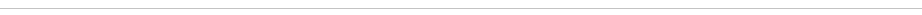 | |
| **37.** | please list some examples of informal training you have had [Question ID: 78578] |
|  | \|  \| \| --- \| |
| 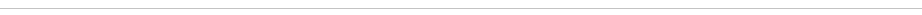 | |
| ***38.** | How important to you is receiving training/professional development on working with people with intellectual disability in a health setting? [Question ID: 78581] |
|  | ●  ○  Not important ○  somewhat important ○  neutral ○  important ○  very important |
|  | **Comments**: |
| 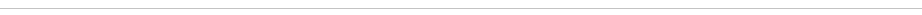 | |
| **39.** | Please comment [Question ID: 78582] |
|  | \|  \| \| --- \| |
|  | **Comments**: |
| 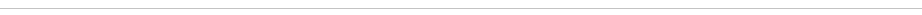 | |
| ***40.** | How important it would be for you to attend training on working with people with intellectual disability? [Question ID: 90673] |
|  | ●  ○  Not important ○  somewhat important ○  neutral ○  important ○  very important |
| 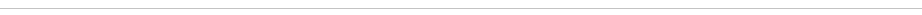 | |
| **41.** | Please Comment [Question ID: 78584] |
|  | \|  \| \| --- \| |
| 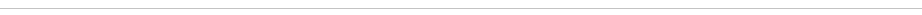 | |
| ***42.** | How supportive would your manager/ educator be in you attending training/professional development on working with people with intellectual disability? [Question ID: 78585] |
|  | ○  Not supportive   ○  somewhat supportive   ○  neutral   ○  supportive   ○  very supportive |
| 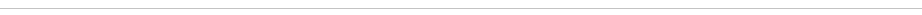 | |
| **43.** | Please Comment [Question ID: 78586] |
|  | \|  \| \| --- \| |
| 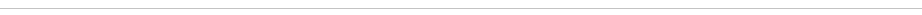 | |
| ***44.** | If intellectual disability education was offered at your place of work, which information or topics would you most like to learn about? (Please tick options most interested) [Question ID: 90674] |
|  | □ What is intellectual disability? □ Characteristics of different diagnosis(eg. Autism, Cerebral Palsy) □ Intellectual disability and communication □ Communication strategies for people with intellectual disability in a health setting □ Intellectual disability and behaviour □ Behavioural strategies for people with intellectual disability in a health setting □ Sensory processing □ Strategies for people with sensory processing difficulties in a health setting □ None of the above/I am not interesting in receiving training □ Other |
|  | **Comments**: |
| 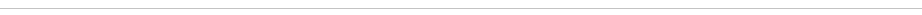 | |
| ***45.** | If an education session/ package were to be introduced for clinical staff, how beneficial would this be to you? [Question ID: 78590] |
|  | ●  ○  not very beneficial ○  somewhat beneficial ○  neutral ○  beneficial ○  very beneficial |
| 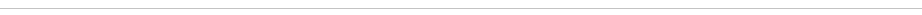 | |
| **46.** | Please comment [Question ID: 78591] |
|  | \|  \| \| --- \| |
| 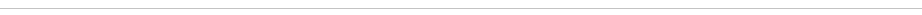 | |
| ***47.** | If strategies and communication aids were to be implemented across your hospital(s) how beneficial would this be to you and your area? (This may include use of photos, visual schedules, social stories of the patient experience and use of IPad etc”) [Question ID: 78592] |
|  | ●  ○  not beneficial ○  somewhat beneficial ○  neutral ○  beneficial ○  very beneficial |
| 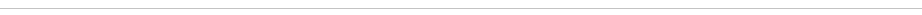 | |
| **48.** | Please comment [Question ID: 78593] |
|  | \|  \| \| --- \| |
| 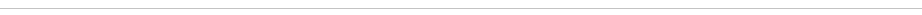 | |
| **49.** | In your professional capacity, can you discuss any care experience you have had with a child and their family with an intellectual disability where delivered interventions may have had a positive or negative impact on their healthcare admission? This question is optional. [Question ID: 78594] |
|  | \|  \| \| --- \| |
| 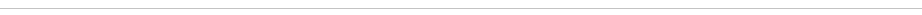 | |
| **50.** | In your experience, what could be improved for a child and their family with an intellectual disability during their hospital admission and subsequent care? This question is optional. [Question ID: 78595] |
|  | \|  \| \| --- \| |
| 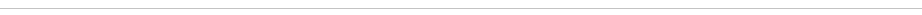 | |
| **51.** | If you would like to speak about your experiences in order to help us understand your responses further, we would like to invite you participate in an online interview with a member of the research team. Please provide us your contact details:  Name  Mobile No  Email Address  Your personal details will not be shared with anyone else outside the research team.  Thank you for your participation. [Question ID: 78596] |
|  | \|  \| \| --- \| |
| 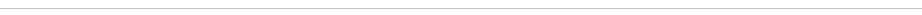 | |

**Post Session Questionnaire for Motivated for Change Program**

| Questionnaire Name |  |
| --- | --- |
| Standard |  |
| Patient Oriented |  |
| Questionnaire Instruction |  |
| Creator Name |  |
| Creator Group |  |

Participant Information: ***Improving the care of children with intellectual disability in hospital (Ethics number: 2020_ETH02240)***

Dear staff,

We are conducting this research survey to better understand your views on your knowledge and skills caring for children and young people with intellectual disability in hospital.

Your responses will assist us in better understanding what we need to change. Your responses will be deidentified and analysed anonymously and not affect your standing in hospital. Any identifying data collected in this survey will be removed and it will be separately stored in a password protected drive within the SCHN, including the details of participants who have consented to participate in an interview.

By completing this survey, you are consenting to participate in this research project. If you have any questions about the project, please email us at natalie.ong@health.nsw.gov.au.

Thank you.

| 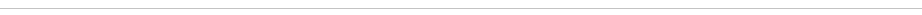 | |
| --- | --- |
| ***1.** | Which hospital(s) are you currently employed by? [Question ID: 86227] |
|  | ●  ○  The Children's Hospital at Westmead ○  Sydney Children's Hospital ○  Both |
|  | **Comments**: |
| 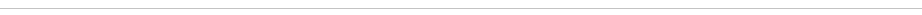 | |
| ***2.** | Please enter your Stafflink Number: (numeric field) [Question ID: 82107] |
|  | \|  \| \| --- \| |
|  | **Comments**: |
| 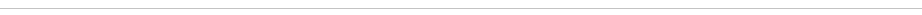 | |
| ***3.** | What is your professional background? [Question ID: 78637] |
|  | ●  ○  Nursing ○  Medical ○  Allied Health ○  Other |
|  | **Comments**: |
| 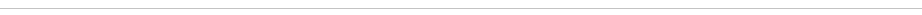 | |
| **4.** | What is your current position? [Question ID: 78506] |
|  | \|  \| \| --- \| |
|  | **Comments**: |
| 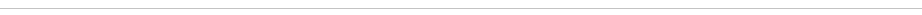 | |
| ***5.** | Which environment best describes your workplace?(Please tick all that apply) [Question ID: 82108] |
|  | □ Triage □ Resus area □ Cubes □ Short Stay □ Observation □ Other |
|  | **Comments**: |
| 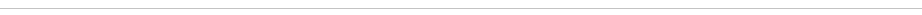 | |
| ***6.** | How many years have you been working in healthcare? [Question ID: 78639] |
|  | ●  ○  0-2 years ○  3-5 years ○  6-10 years ○  11-14 years ○  +15 years |
|  | **Comments**: |
| 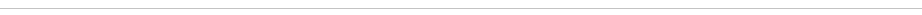 | |
| ***7.** | On average, how much professional contact do you have with people who have an  intellectual disability? [Question ID: 78640] |
|  | ●  ○  None ○  Daily ○  weekly ○  monthly ○  yearly |
|  | **Comments**: |
| 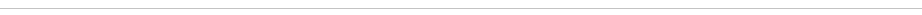 | |
| ***8.** | On average, how much non-professional contact do you have with people with intellectual disabilities? [Question ID: 78641] |
|  | ●  ○  None ○  daily ○  weekly ○  monthly ○  yearly |
|  | **Comments**: |
| 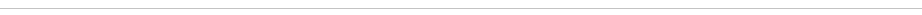 | |
| ***9.** | . Please rate: Overall how successful do you feel have these interactions with people with intellectual disability been (professional and non- professional)? Where 1 is very poor up to 10 Very successful (numeric field with 10 decimal point.) [Question ID: 78642] |
|  | \|  \| \| --- \|   *(1)* |
|  | **Comments**: |
| 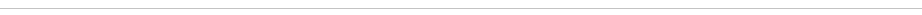 | |
| ***10.** | I have participated in a intellectual disability training session. [Question ID: 107810] |
|  | ●  ○  Completed the Motivated for Change Program ○  Partially completed the Motivated for Change Program ○  Not completed any ○  Other training, please specify below |
|  | **Comments**: |
| 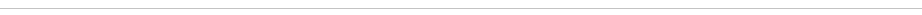 | |
| **11.** | Please Specify: [Question ID: 107869] |
|  | \|  \| \| --- \| |
|  | **Comments**: |
| 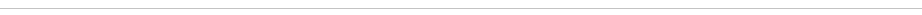 | |
| ***12.** | I feel I have the necessary knowledge, training and skills to manage successfully with people with an intellectual disability in my healthcare environment. [Question ID: 108350] |
|  | ●  ○  Strongly disagee ○  Disagree ○  Neutral ○  Agree ○  Strongly Agree |
|  | **Comments**: |
| 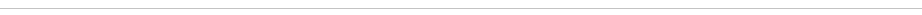 | |
| ***13.** | I feel I have the necessary knowledge, training and skills to manage successfully people with a range of communication skills and difficult behaviours in my healthcare environment. [Question ID: 108351] |
|  | ●  ○  Strongly Disagree ○  Disagree ○  Neutral ○  Agree ○  Strongly agree |
|  | **Comments**: |
| 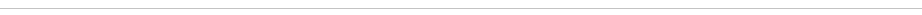 | |
| ***14.** | I believe that treating people with intellectual disability is a part of my role/s as a health professional. [Question ID: 108352] |
|  | ●  ○  Strongly Disagree ○  Disagree ○  Neutral ○  Agree ○  Strongly Agree |
|  | **Comments**: |
| 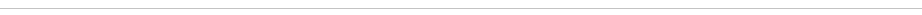 | |
| ***15.** | I believe that treating people with intellectual disability is an expectation from my colleagues and my professional body. [Question ID: 108353] |
|  | ●  ○  Strongly Disagree ○  Disagree ○  Neutral ○  Agree ○  Strongly agree |
|  | **Comments**: |
| 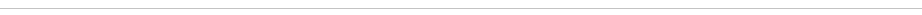 | |
| ***16.** | The physical and mental health needs of people with intellectual disability can be adequately met in a mainstream health service [Question ID: 108354] |
|  | ●  ○  Strongly disagree ○  Disagree ○  Neutral ○  Agree ○  Strongly Agree |
|  | **Comments**: |
| 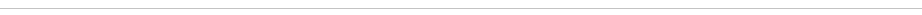 | |
| ***17.** | My knowledge, training and skills are easily implemented in my healthcare practice and work environment when I contact patients with intellectual disability. [Question ID: 108355] |
|  | ●  ○  Strongly Disagree ○  Disagree ○  Neutral ○  Agree ○  Strongly Agree |
|  | **Comments**: |
| 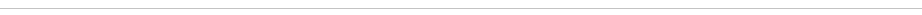 | |
| ***18.** | The physical and mental health of people with intellectual disability is no better or worse than people without intellectual disability. [Question ID: 108356] |
|  | ●  ○  Strongly Disagree ○  Disagree ○  Neutral ○  Agree ○  Strongly Agree |
|  | **Comments**: |
| 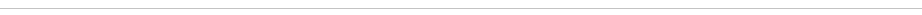 | |
| ***19.** | Even if it is hard to understand people with intellectual disability, I think it is still important to talk to them personally [Question ID: 108357] |
|  | ●  ○  Strongly disagree ○  Disagree ○  Neutral ○  Agree ○  Strongly Agree |
|  | **Comments**: |
| 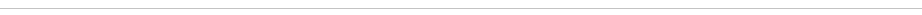 | |
| ***20.** | I would not be able to spend time and explain treatment plans to a person with intellectual disability as they did not understand [Question ID: 108359] |
|  | ●  ○  Strongly Disagree ○  Disagree ○  Neutral ○  Agree ○  Strongly Agree |
|  | **Comments**: |
| 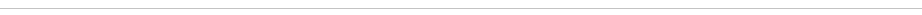 | |
| ***21.** | I would like to have information given to me about the patient with an intellectual disability who will use my health services during their admission/treatment [Question ID: 108358] |
|  | ●  ○  Strongly Disagree ○  Disagree ○  Neutral ○  Agree ○  Strongly Agree |
|  | **Comments**: |
| 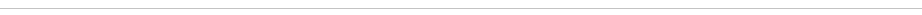 | |
| ***22.** | When working with a patient who has an intellectual disability, I make time to ensure that I am clear on what their health difficulty is, even if that means I run over schedule [Question ID: 108360] |
|  | ●  ○  Strongly Disagree ○  Disagree ○  Neutral ○  Agree ○  Strongly Agree |
|  | **Comments**: |
| 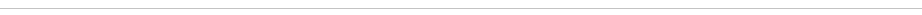 | |
| ***23.** | Carer/family members/relatives are a vital resource in providing information and support about the person with an intellectual disability. Even if a patient with an intellectual disability has good communication skills, I still find it more useful to talk to the carer first about their illness rather than the child or the young person with intellectual disability. [Question ID: 108361] |
|  | ●  ○  Strongly Disagree ○  Disagree ○  Neutral ○  Agree ○  Strongly Agree |
|  | **Comments**: |
| 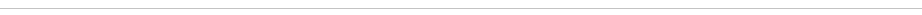 | |
| ***24.** | I always try and explore the health complaints of a patient who has an intellectual disability by communicating with the patient themselves, than solely asking their carer. [Question ID: 108362] |
|  | ●  ○  Strongly Disagree ○  Disagree ○  Neutral ○  Agree ○  Strongly Agree |
|  | **Comments**: |
| 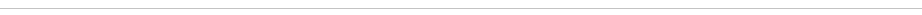 | |
| ***25.** | Please rate your knowledge, skills and experience working with people with development disability [Question ID: 82109] |
|  | \|  \| very low \| low \| neutral \| high \| very high \| \| --- \| --- \| --- \| --- \| --- \| --- \| \| Knowledge \| ○ \| ○ \| ○ \| ○ \| ○ \| \| Skills and level of experience \| ○ \| ○ \| ○ \| ○ \| ○ \| |
|  | **Comments**: |
| 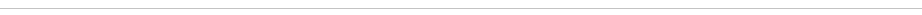 | |
| ***26.** | Please rate your knowledge, skills and experience in working with people with a physical disability [Question ID: 82110] |
|  | \|  \| very low \| low \| neutral \| high \| very high \| \| --- \| --- \| --- \| --- \| --- \| --- \| \| Knowledge \| ○ \| ○ \| ○ \| ○ \| ○ \| \| Skills and Level of experience \| ○ \| ○ \| ○ \| ○ \| ○ \| |
|  | **Comments**: |
| 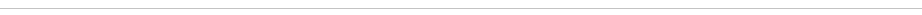 | |
| ***27.** | Please rate your knowledge, skills and experience in working with people with an intellectual disability and challenging behaviours [Question ID: 82111] |
|  | \|  \| very low \| low \| neutral \| high \| very high \| \| --- \| --- \| --- \| --- \| --- \| --- \| \| Knowledge \| ○ \| ○ \| ○ \| ○ \| ○ \| \| Skills and level of Experience \| ○ \| ○ \| ○ \| ○ \| ○ \| |
|  | **Comments**: |
| 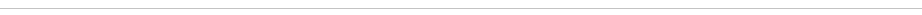 | |
| ***28.** | Please rate your knowledge, skills and experience in working with communication difficulties experienced by people with intellectual disabilities [Question ID: 82112] |
|  | \|  \| very low \| low \| neutral \| high \| very high \| \| --- \| --- \| --- \| --- \| --- \| --- \| \| Knowledge \| ○ \| ○ \| ○ \| ○ \| ○ \| \| Skills and level of experience \| ○ \| ○ \| ○ \| ○ \| ○ \| |
|  | **Comments**: |
| 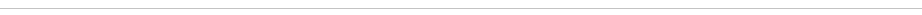 | |
| ***29.** | Please rate your knowledge, skills and experience in working with strategies used to support the communication of people with intellectual disabilities (e.g. Picture boards, signing, social stories, picture timetables) [Question ID: 82113] |
|  | \|  \| very low \| low \| neutral \| high \| very high \| \| --- \| --- \| --- \| --- \| --- \| --- \| \| Knowledge \| ○ \| ○ \| ○ \| ○ \| ○ \| \| Skills and level of experience \| ○ \| ○ \| ○ \| ○ \| ○ \| |
|  | **Comments**: |
| 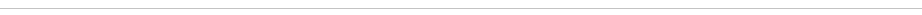 | |
| ***30.** | Please rate your knowledge, skills and experience in working with triggers and causes of challenging behavioural issues in people with intellectual disabilities [Question ID: 82115] |
|  | \|  \| very low \| low \| neutral \| high \| very high \| \| --- \| --- \| --- \| --- \| --- \| --- \| \| Knowledge \| ○ \| ○ \| ○ \| ○ \| ○ \| \| Skills and level of experience \| ○ \| ○ \| ○ \| ○ \| ○ \| |
|  | **Comments**: |
| 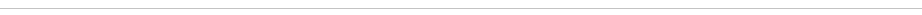 | |
| ***31.** | Please rate your knowledge, skills and experience in working with strategies to manage challenging behaviours in people with intellectual disabilities in terms of tome constraints of being a healthcare worker. [Question ID: 82114] |
|  | \|  \| very low \| low \| neutral \| high \| very high \| \| --- \| --- \| --- \| --- \| --- \| --- \| \| Knowledge \| ○ \| ○ \| ○ \| ○ \| ○ \| \| Skills and level of experience \| ○ \| ○ \| ○ \| ○ \| ○ \| |
|  | **Comments**: |
| 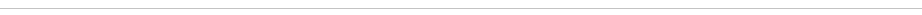 | |
| ***32.** | Please identify what you feel are contributing causes of difficulties experienced by patients and their families with an intellectual disability during their hospital admission and subsequent care. You may indicate more than one point. [Question ID: 78666] |
|  | □ communication □ unable to understand □ frustration from communication difficulties □ unrealistic expectations □ physical limitations □ emotional regulation □ sensory stimulation □ unwanted personal/physical contact with staff □ challenging behaviours □ ritualistic behaviour □ compulsive behaviour □ new environment □ time constraints of healthcare woprker □ adjustment to ward routine(s) □ environmental stimulation such as noise □ environmental stimulation such as busy ward areas □ none of the above □ Other |
|  | **Comments**: |
| 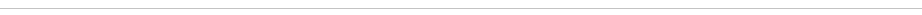 | |
| ***33.** | Are you aware of the various policies?     [Question ID: 78667] |
|  | \|  \| yes \| no \| \| --- \| --- \| --- \| \| NSW health Policy Directive, Disability-People with disabilities:responding to their needs during Hospitalisation \| ○ \| ○ \| \| Pharmacological strategies for children with developmental disability during investigations/procedures \| ○ \| ○ \| \| Developmental Disability Patients – Acute Management Plan Flowchart \| ○ \| ○ \| \| Non-restrictive Care for Mental Health Paediatric Inpatients with Co-morbid Intellectual Disability and/or Autism Spectrum Disorder \| ○ \| ○ \| |
|  | **Comments**: |
| 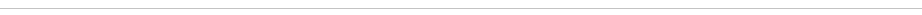 | |
| ***34.** | Have you read or accessed the " any of the policies above. Please indicate which one s below: [Question ID: 82116] |
|  | □ 1. NSW health Policy Directive, Disability-People with disabilities:Responding to their needs during Hospitalisation □ 2. Pharmacological strategies for children with developmental disability during investigations/procedures □ 3. Developmental Disability Patients – Acute Management Plan Flowchart □ 4. Non-restrictive Care for Mental Health Paediatric Inpatients with Co-morbid Intellectual Disability and/or Autism Spectrum Disorder |
|  | **Comments**: |
| 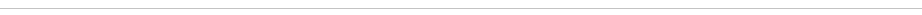 | |
| ***35.** | To the best of your knowledge, do you feel these policies have been implemented in your care setting? [Question ID: 78669] |
|  | ○ Yes ○ No |
|  | **Comments**: |
| 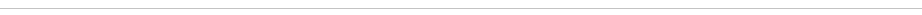 | |
| ***36.** | If strategies and communication aids were to be implemented across your hospital(s) how beneficial would this be to you and your area? (This may include use of photos, visual schedules, social stories of the patient experience and use of IPad etc”) [Question ID: 108363] |
|  | ●  ○  not beneficial ○  somewhat beneficial ○  neutral ○  beneficial ○  very beneficial |
|  | **Comments**: |
| 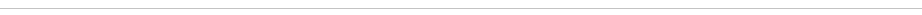 | |
| **37.** | In your professional capacity, can you discuss any care experience you have had with a child and their family with an intellectual disability where delivered interventions may have had a positive or negative impact on their healthcare admission? [Question ID: 107812] |
|  | \|  \| \| --- \| |
|  | **Comments**: |
| 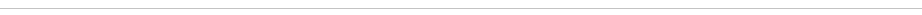 | |
| **38.** | In your experience, what could be improved for a child and their family with an intellectual disability during their hospital admission and subsequent care? [Question ID: 107813] |
|  | \|  \| \| --- \| |
|  | **Comments**: |
| 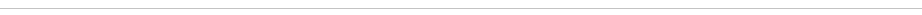 | |
| **39.** | If you would like to speak about your experiences in order to help us understand your responses further, we would like to invite you participate in an online interview with a member of the research team. Please provide us your contact details:  Name Mobile No Email Address   Your personal details will not be shared with anyone else outside the research team.   Thank you for your participation! [Question ID: 107665] |
|  | \|  \| \| --- \| |
|  | **Comments**: |
| 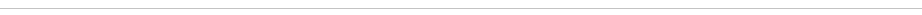 | |

  
 

**Aggregated Survey Results (n=131/89)**

**Figure 1**

Average levels of knowledge, skills and experience in working with children and young people with intellectual and developmental disability, along with error bars.

**Figure 2**

Average levels of knowledge and skills in working with children and young people with physical disabilities, along with error bars

**Figure 3**

Average levels of knowledge, skills and experience in working with children and young people with intellectual and developmental disabilities and challenging behaviours, along with error bars

**Figure 4**

Average levels of knowledge, skills and experience in working with communication difficulties of children and young people with intellectual and developmental disabilities, along with error bars, along with error bars

**Figure 5**

Average levels of knowledge and skills in working with communication support strategies for individuals with intellectual disabilities, along with error bars

**Figure 6**

Average levels of knowledge and skills in working with triggers and challenging behaviours in children and young people with intellectual and developmental disabilities, along with error bars

**Figure 7**

Average levels of knowledge and skills in strategies to manage challenging behaviours in children and young people with intellectual and developmental disabilities, along with error bars

**Paired survey results**

**Table 1: Results of the Wilcoxon test for subjective indicators of knowledge and competence of staff before and after training (n=39)**

|  | Pretest | | | Posttest | | |  |  |  | |  |  |
| --- | --- | --- | --- | --- | --- | --- | --- | --- | --- | --- | --- | --- |
| Dependent variable | | *Mdn* | *IQR* | | *Mdn* | *IQR* | *Z* | *p* | | *r* | *p_adj._* |  |
| **Working with children and young people with intellectual and developmental disability** | |  |  | |  |  |  |  | |  |  |  |
| Knowledge | | 3,00 | 1,00 | | 4,00 | 1,00 | -3,40 | **<0,001** | | 0,38 | **0,007** |  |
| Skills and Level of experience | | 3,00 | 1,00 | | 4,00 | 1,00 | -3,44 | **<0,001** | | 0,39 | **0,007** |  |
| **Working with children and young people with physical disability** | |  |  | |  |  |  |  | |  |  |  |
| Knowledge | | 3,00 | 1,00 | | 4,00 | 0,00 | -3,66 | **<0,001** | | 0,41 | **0,007** |  |
| Skills and Level of experience | | 4,00 | 1,00 | | 4,00 | 1,00 | -2,78 | **0,005** | | 0,31 | 0,070 |  |
| **Working with children and young people with intellectual and developmental disability and challenging behaviours** | |  |  | |  |  |  |  | |  |  |  |
| Knowledge | | 3,00 | 2,00 | | 4,00 | 1,00 | -2,63 | **0,008** | | 0,30 | 0,112 |  |
| Skills and Level of experience | | 3,00 | 2,00 | | 4,00 | 1,00 | -3,09 | **0,002** | | 0,35 | **0,028** |  |
| **Working with communication difficulties experienced by children and young people with intellectual and developmental disability** | |  |  | |  |  |  |  | |  |  |  |
| Knowledge | | 3,00 | 1,00 | | 4,00 | 1,00 | -4,54 | **<0,001** | | 0,51 | **0,007** |  |
| Skills and Level of experience | | 3,00 | 1,00 | | 4,00 | 1,00 | -3,99 | **<0,001** | | 0,45 | **0,007** |  |
| **Working with strategies used to support the communication of children and young people with intellectual and developmental disability** | |  |  | |  |  |  |  | |  |  |  |
| Knowledge | | 3,00 | 1,00 | | 4,00 | 1,00 | -4,25 | **<0,001** | | 0,48 | **0,007** |  |
| Skills and Level of experience | | 3,00 | 1,00 | | 3,00 | 1,00 | -3,49 | **<0,001** | | 0,40 | **0,007** |  |
| **Working with triggers and causes of challenging behavioural issues in children and young people with intellectual and developmental disability** | |  |  | |  |  |  |  | |  |  |  |
| Knowledge | | 3,00 | 1,00 | | 4,00 | 1,00 | -3,46 | **<0,001** | | 0,39 | **0,007** |  |
| Skills and Level of experience | | 3,00 | 1,00 | | 3,00 | 1,00 | -3,54 | **<0,001** | | 0,40 | **0,007** |  |
| **Working with strategies to manage challenging behaviours in children and young people with intellectual and developmental disability** | |  |  | |  |  |  |  |  | |  |  |
| Knowledge | | 3,00 | 1,00 | | 3,00 | 1,00 | -3,52 | **<0,001** | 0,40 | | **0,007** |  |
| Skills and Level of experience | | 3,00 | 1,00 | | 3,00 | 1,00 | -2,65 | **0,008** | 0,30 | | 0,112 |  |

*Annotation: n - group size; Mdn - median; IQR - interquartile range; Z - test statistic value; p - statistical significance; r - effect size indicator;* *p_adj_. – Bonferroni corrected p-value.*

The result of the Wilcoxon test for all introduced pairs of variables turned out to be statistically significant, indicating that the subjectively assessed level of knowledge, skills, and experience of the personnel significantly changed. Furthermore, the negative value of the Z test statistic for each pair of tested variables indicates that the average ratings of hospital staff increased after the workshops. They rated their knowledge, skills, and level of experience better in the overall care of patients with developmental, physical, and intellectual disabilities. Additionally, they rated their knowledge and skills better in dealing with communication issues in such patients, as well as their problematic behaviours. In most cases, the effect sizes were moderate, and in one case, the effect was strong.

A Bonferroni correction was applied to p-values in order to control for number of comparisons. P-values below 0,001 were calculated as 0,0005. After applying the correction 11 out of 14 tests were statistically significant, which indicated good reliability of the results, given high conservativeness of the Bonferroni correction.

**Level of knowledge and skills before and after training**

Further analysis was conducted to determine the changes is level in the level of knowledge and skills due educational training amongst doctors and nurses. For this purpose, the Wilcoxon test for paired observations was conducted, divided into groups. The results are presented in Tables 3 and 4.

**Table 2**

**Level of knowledge and skills in working with** **children and young people with intellectual disability before and after training, among doctors**

|  | Pretest | | | Posttest | | |  | |  | |  |  |
| --- | --- | --- | --- | --- | --- | --- | --- | --- | --- | --- | --- | --- |
| Dependent variable | *Mdn* | *IQR* | *Mdn* | | *IQR* | *Z* | | *p* | | *r* | | *p_adj._* |
| **Working with children and young people with intellectual and developmental disability** |  |  |  | |  |  | |  | |  | |  |
| Knowledge | 3,00 | 0,00 | 3,00 | | 1,00 | -1,73 | | 0,083 | | 0,31 | | 1,000 |
| Skills and Level of experience | 3,00 | 1,00 | 3,00 | | 1,00 | -2,07 | | **0,038** | | 0,37 | | 0,532 |
| **Working with children and young people with physical disability** |  |  |  | |  |  | |  | |  | |  |
| Knowledge | 3,00 | 1,00 | 4,00 | | 1,00 | -2,33 | | **0,020** | | 0,41 | | 0,280 |
| Skills and Level of experience | 3,00 | 1,00 | 3,50 | | 1,00 | -1,61 | | 0,107 | | 0,28 | | 1,000 |
| **Working with children and young people with intellectual and developmental disability and challenging behaviours** |  |  |  | |  |  | |  | |  | |  |
| Knowledge | 3,00 | 1,00 | 3,00 | | 1,00 | -1,87 | | 0,061 | | 0,33 | | 0,854 |
| Skills and Level of experience | 3,00 | 1,00 | 3,00 | | 1,00 | -2,50 | | **0,013** | | 0,44 | | 0,182 |
| **Working with communication difficulties experienced by children and young people with intellectual and developmental disability** |  |  |  | |  |  | |  | |  | |  |
| Knowledge | 3,00 | 1,00 | 3,00 | | 1,00 | -3,13 | | **0,002** | | 0,55 | | **0,028** |
| Skills and Level of experience | 3,00 | 1,00 | 3,00 | | 1,00 | -2,65 | | **0,008** | | 0,47 | | 0,112 |
| **Working with strategies used to support the communication of children and young people with intellectual and developmental disability** |  |  |  | |  |  | |  | |  | |  |
| Knowledge | 2,00 | 1,00 | 4,00 | | 1,00 | -2,99 | | **0,003** | | 0,53 | | **0,042** |
| Skills and Level of experience | 2,00 | 1,00 | 3,00 | | 1,00 | -3,04 | | **0,002** | | 0,54 | | **0,028** |
| **Working with triggers and causes of challenging behavioural issues in children and young people with intellectual and developmental disability** |  |  |  | |  |  | |  | |  | |  |
| Knowledge | 3,00 | 1,00 | 3,00 | | 1,00 | -2,65 | | **0,008** | | 0,47 | | 0,112 |
| Skills and Level of experience | 3,00 | 1,00 | 3,00 | | 0,75 | -2,50 | | **0,013** | | 0,44 | | 0,182 |
| **Working with strategies to manage challenging behaviours in children and young people with intellectual and developmental disability** |  |  |  | |  |  | |  | |  | |  |
| Knowledge | 2,50 | 1,00 | 3,00 | | 0,00 | -2,33 | | **0,020** | | 0,41 | | 0,280 |
| Skills and Level of experience | 2,50 | 1,00 | 3,00 | | 1,00 | -1,81 | | 0,070 | | 0,32 | | 0,980 |

*Annotation: n - group size; Mdn - median; IQR - interquartile range; Z - test statistic value; p - statistical significance; r - effect size indicator; p_adj_. – Bonferroni corrected p-value.*

Among doctors, statistically significant changes were observed in 10 out of 14 pairs of variables studied. In all cases, doctors achieved higher scores in the post-test, as indicated by the negative value of the Z test statistic. These effects were ranged from moderate to strong.
After applying Bonferroni correction 3 out of 10 tests were statistically significant, which indicated high reliability of these results.

**Table 3**

**Level of knowledge and skills in working with children and young people with intellectual disability before and after training, among nurses.**

|  | Pretest | | Posttest | |  |  |  |  |
| --- | --- | --- | --- | --- | --- | --- | --- | --- |
| Dependent variable | *Mdn* | *IQR* | *Mdn* | *IQR* | *Z* | *p* | *r* | *p_adj._* |
| **Working with people with development disability** |  |  |  |  |  |  |  |  |
| Knowledge | 3,00 | 0,50 | 4,00 | 1,00 | -3,46 | **<0,001** | 0,53 | **0,007** |
| Skills and Level of experience | 3,00 | 0,50 | 4,00 | 1,00 | -3,00 | **0,003** | 0,46 | **0,042** |
| **Working with people with physical disability** |  |  |  |  |  |  |  |  |
| Knowledge | 3,00 | 1,00 | 4,00 | 0,00 | -2,84 | **0,005** | 0,44 | 0,070 |
| Skills and Level of experience | 4,00 | 1,00 | 4,00 | 0,00 | -2,50 | **0,013** | 0,39 | 0,182 |
| **Working with people with intellectual disability and challenging behaviours** |  |  |  |  |  |  |  |  |
| Knowledge and skills | 3,00 | 0,50 | 4,00 | 1,00 | -2,05 | **0,040** | 0,32 | 0,560 |
| Skills and Level of experience | 3,00 | 1,00 | 4,00 | 1,00 | -1,81 | 0,071 | 0,28 | 0,994 |
| **Working with communication difficulties experienced by people with intellectual disabilities** |  |  |  |  |  |  |  |  |
| Knowledge and skills | 3,00 | 1,00 | 4,00 | 0,50 | -3,15 | **0,002** | 0,49 | **0,028** |
| Skills and Level of experience | 3,00 | 1,00 | 4,00 | 1,00 | -2,83 | **0,005** | 0,44 | 0,070 |
| **Working with strategies used to support the communication of people with intellectual disabilities** |  |  |  |  |  |  |  |  |
| Knowledge | 3,00 | 1,50 | 4,00 | 1,00 | -3,22 | **0,001** | 0,50 | **0,014** |
| Skills and Level of experience | 3,00 | 1,00 | 3,00 | 1,50 | -2,15 | **0,031** | 0,33 | 0,434 |
| **Working with triggers and causes of challenging behavioural issues in people with intellectual disabilities** |  |  |  |  |  |  |  |  |
| Knowledge | 3,00 | 0,50 | 4,00 | 1,00 | -2,81 | **0,005** | 0,43 | 0,070 |
| Skills and Level of experience | 3,00 | 1,00 | 4,00 | 1,00 | -2,81 | **0,005** | 0,43 | 0,070 |
| **Working with strategies to manage challenging behaviours in people with intellectual disabilities** |  |  |  |  |  |  |  |  |
| Knowledge | 3,00 | 1,00 | 3,00 | 1,00 | -3,46 | **<0,001** | 0,53 | **0,007** |
| Skills and Level of experience | 3,00 | 1,50 | 3,00 | 1,00 | -2,71 | **0,007** | 0,42 | 0,098 |

*Annotation: n - group size; Mdn - median; IQR - interquartile range; Z - test statistic value; p - statistical significance; r - effect size indicator; p_adj_. – Bonferroni corrected p-value.*

For nurses, statistically significant results were observed in 13 out of 14 pairs of variables studied. In all cases, nurses achieved higher scores in the post-test, as indicated by the negative value of the Z test statistic. These effects were moderate to strong.

The relationship between the sense of competence and the level of knowledge with work experience and the frequency of contacts, both professional and personal, with disabled patients was examined. Spearman's rho correlation analysis was conducted, divided into doctors and nurses. The results are presented in Tables 1 and 2.
After applying Bonferroni correction 5 out of 13 tests were statistically significant, which indicated high reliability of these results.

**Table 4**

**Correlations between work experience, frequency of contact with disabled patients, sense of competence, and level of knowledge and skills in working with disabled patients among doctors.**
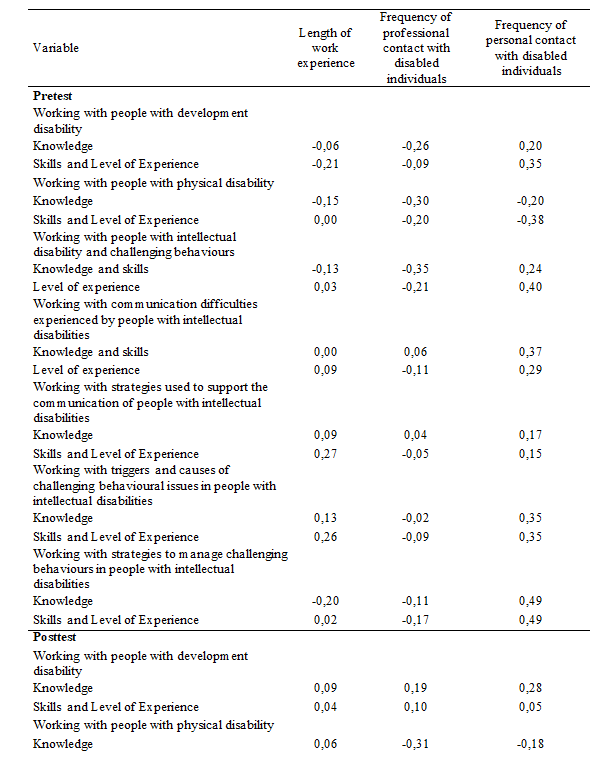


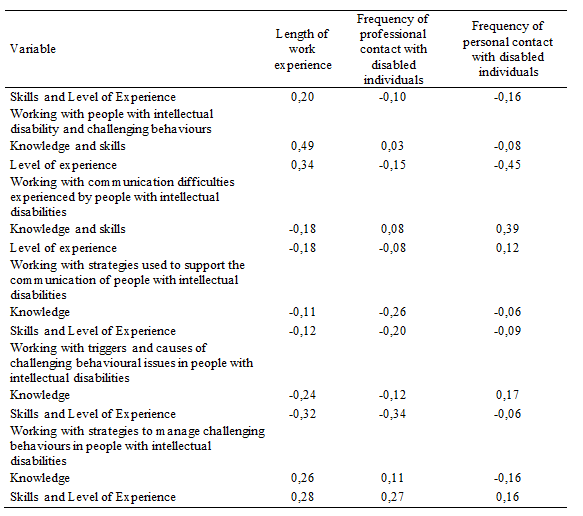


**Table 5**

Correlations between work experience, frequency of contact with disabled patients, sense of competence, and level of knowledge and skills in working with disabled patients among nurses.

| Variable | Length of work experience | Frequency of professional contact with disabled individuals | Frequency of personal contact with disabled individuals |
| --- | --- | --- | --- |
| **Pretest** |  |  |  |
| Working with people with development disability |  |  |  |
| Knowledge | 0,18 | 0,40 | 0,41 |
| Skills and Level of Experience | 0,02 | **0,65^**^** | 0,10 |
| Working with people with physical disability |  |  |  |
| Knowledge | -0,18 | 0,25 | 0,13 |
| Skills and Level of Experience | -0,03 | 0,32 | 0,36 |
| Working with people with intellectual disability and challenging behaviours |  |  |  |
| Knowledge and skills | -0,04 | 0,40 | 0,05 |
| Level of experience | -0,34 | **0,46^*^** | 0,04 |
| Working with communication difficulties experienced by people with intellectual disabilities |  |  |  |
| Knowledge and skills | 0,16 | 0,36 | 0,39 |
| Level of experience | 0,31 | **0,49^*^** | 0,39 |
| Working with strategies used to support the communication of people with intellectual disabilities |  |  |  |
| Knowledge | 0,07 | 0,01 | 0,31 |
| Skills and Level of Experience | -0,02 | 0,02 | 0,42 |
| Working with triggers and causes of challenging behavioural issues in people with intellectual disabilities |  |  |  |
| Knowledge | 0,07 | 0,26 | **0,69^***^** |
| Skills and Level of Experience | 0,08 | 0,37 | 0,34 |
| Working with strategies to manage challenging behaviours in people with intellectual disabilities |  |  |  |
| Knowledge | -0,08 | 0,30 | 0,21 |
| Skills and Level of Experience | -0,17 | 0,23 | 0,01 |
| **Posttest** |  |  |  |
| Working with people with development disability |  |  |  |
| Knowledge | 0,05 | 0,08 | 0,23 |
| Skills and Level of Experience | 0,11 | 0,11 | 0,21 |
| Working with people with physical disability |  |  |  |
| Knowledge | 0,20 | 0,16 | 0,00 |
| Skills and Level of Experience | 0,30 | 0,13 | 0,15 |
| Working with people with intellectual disability and challenging behaviours |  |  |  |
| Knowledge and skills | -0,14 | 0,23 | 0,07 |
| Level of experience | 0,01 | 0,23 | 0,14 |
| Working with communication difficulties experienced by people with intellectual disabilities |  |  |  |
| Knowledge and skills | 0,07 | 0,00 | 0,24 |
| Level of experience | 0,11 | 0,02 | 0,22 |
| Working with strategies used to support the communication of people with intellectual disabilities |  |  |  |
| Knowledge | 0,12 | -0,11 | 0,10 |
| Skills and Level of Experience | 0,09 | -0,07 | 0,01 |
| Working with triggers and causes of challenging behavioural issues in people with intellectual disabilities |  |  |  |
| Knowledge | 0,03 | 0,16 | 0,35 |
| Skills and Level of Experience | 0,10 | 0,19 | 0,34 |
| Working with strategies to manage challenging behaviours in people with intellectual disabilities |  |  |  |
| Knowledge | -0,04 | 0,20 | 0,28 |
| Skills and Level of Experience | -0,04 | 0,20 | 0,28 |

^***^ - *p* < 0,001; ^**^ - *p* < 0,01; ^*^ - *p* < 0,05

Among doctors, no significant correlations were observed between the variables under investigation. Among nurses, statistically significant correlations were observed between the variables, but only in the pre-training measurement. The frequency of contact with disabled patients was significantly positively correlated with the level of knowledge and skills in some areas of work. The strengths of these relationships were moderate to strong.

**Awareness of policies regarding children and young people with intellectual or developmental disabilities before and after educational training**

The next step of the analysis examined whether the educational training impacted the level of awareness of selected policies regarding the treatment and hospitalization of patients with disabilities. Cross-tabulation tables with Table **6**

| For Doctors | Pretest | Posttest | |  |
| --- | --- | --- | --- | --- |
| Dependent variable |  | Yes | No | *p* |
| NSW health Policy Directive, Disability-People with disabilities: responding to their needs during Hospitalisation | Yes | 1 | 1 | 0,070 |
|  | No | 7 | 7 |  |
| Pharmacological strategies for children with developmental disability during investigations/procedures | Yes | 3 | 0 | **0,004** |
|  | No | 9 | 4 |  |
| Developmental Disability Patients – Acute Management Plan Flowchart | Yes | 1 | 1 | 0,070 |
|  | No | 7 | 7 |  |
| Non-restrictive Care for Mental Health Paediatric Inpatients with Co-morbid Intellectual Disability and/or Autism Spectrum Disorder | Yes | 0 | 0 | **0,016** |
|  | No | 7 | 9 |  |
| For Nurses | Pretest | Posttest | |  |
| Dependent variable |  | Yes | No | *p* |
| NSW health Policy Directive, Disability-People with disabilities: responding to their needs during Hospitalisation | Yes | 4 | 1 | **0,003** |
|  | No | 12 | 4 |  |
| Pharmacological strategies for children with developmental disability during investigations/procedures | Yes | 1 | 1 | **0,003** |
|  | No | 12 | 7 |  |
| Developmental Disability Patients – Acute Management Plan Flowchart | Yes | 3 | 0 | **<0,001** |
|  | No | 16 | 2 |  |
| Non-restrictive Care for Mental Health Paediatric Inpatients with Co-morbid Intellectual Disability and/or Autism Spectrum Disorder | Yes | 3 | 0 | **0,002** |
|  | No | 10 | 8 |  |

Among doctors, statistically significant changes were observed in the distribution of two variables. Among nurses, changes were observed in all the variables studied.

**Association of work experience, frequency of contact with children and young people with intellectual and developmental disability, and level of knowledge and skills**

The relationship between the sense of competence and the level of knowledge with work experience and the frequency of contacts, both professional and personal, with disabled patients was examined using Spearman's rho correlation analysis separately for doctors and nurses.

Among doctors, no significant correlations were observed between the variables under investigation. However, among nurses, statistically significant correlations were observed in the pre-training variable. Specifically, the frequency of contact with children and young people with intellectual and developmental disability was significantly positively correlated with the level of knowledge and skills in some areas of work. The strengths of these relationships were moderate to strong, with no correlation in the post- test scores suggesting a flattening effect of self- perceived knowledge, skills and experience after completion of the program
