# Supplementary material for: Improving the Safety and Quality of Care for Children and Young People With Intellectual and Developmental Disability. The Motivated for Change Programme in a Paediatric Emergency Department Setting. A Mixed Methods Study
Source: Health Expect. 2026 Aug 2;29(4):e70776. doi: 10.1111/hex.70776 (PMC13429100; doi:10.1111/hex.70776)
Supplement: Supplementary file 2 — Supporting File 2 [file HEX-29-e70776-s002.docx]

**Supplementary Data 2:**

1. **Quotes from the pre and post intervention phase**

| **Pre intervention** |  |
| --- | --- |
| **Discriminating against disability** | *Because you feel that because she had the disability, she was being discriminated against for getting appropriate healthcare. If she had presented as a normal person, she would have probably had a different path. Isn't that? Is that what we're trying to say?. Staff 1* |
| **Environmental Factors** | *On this second presentation was again hit by the noise in the unit and how loud it is and how constant it is. Observation (Obs) 3*  *One nurse made comment to observer that there is no real quiet area in department but try and use single rooms. Obs 5*  *Obviously, it’s not a really nice environment to stay in. There’s a lot of lights. It’s very loud. Kids are in pain. Parents are stressed. So, you can’t expect to have a nice bed and a cocktail and a quiet place for yourself [laughs]. It’s emergency services, so it’s not a fun place to be. Parent 1*  *With this particular child, mum was quite agitated as well. Mum felt like - he was put in the mental health room, the SARS room, which I'm not sure if you've seen that. It's not a nice room. Staff 2* |
| **Process Factors**  Identifying child can be challenging | *From sight I can see that there is something, there is something going on developmentally, even though it was not disclosed afterwards. After the procedures were put finished. Staff 3*  *Yeah, that's probably what it is. It's usually and I don't know, it's just like you got feeling sometimes and you can't put a finger on it. Staff 4*  *You can’t sleep yourself. Your kid can’t sleep. You’re stressed. You’re tired . Typically, those days have been really long at the emergency downstairs. Parent 1*  *Waiting area – Nurse engaging with child – bent to her level, talking to child, giving choice – are you ok? Do you need anything?*  *Staff alerting to another staff mothers needs – Nurse strolling the waiting area eyeballing families and children.*  *Nurse explaining to father long wait times and she will check and update him. Then also advised other parents re wait times. Obs 6* |
| **Staff - Child interaction inconsistent** | *It was really complex and difficult. Yeah, on the stress thing, yeah. Staff 1*  *You know, and then you sometimes are worried that if they're gonna scream or, you know, react and then like the children in the rest of the ward, how they're going to kind of interpret that kind of behaviour if they've seen behaviour of, like, these kids biting themselves or…Staff 4*  *Child observed to be still very upset and was being comforted by his parents. Obs 5*  *I don't know probably that I think that needs to be.. sort of tailored for each one of them depending on their on their likes and interests. Staff 1*  *Observed Doctor communication with parents, didn’t interact with child. Obs 2*  *… because we don't know how they communicate. Staff 4*  *Nurse observed undertaking the taking of a child’s temperature and to help settle the child was observed to interact with the child in a tickling game which seemed to put the child at ease. Obs 3*  *[About the Child Life Therapist] Yeah, they give us a lot of colouring and activities, … for special need kids, but in general…..they're very dedicated and they, you know, try to communicate with the parents and try to find out certain things that can put [in place]. Staff 1* |
| **Staff - Parent Interaction inconsistent** | *Mum stated that no one is listening to her concerns or willing to help her Obs 10.*  *Nurse then ended with acknowledging mothers concerns and that she would put a note in system but still a very long wait as child was well. She tried to redirect back to a community pathway but mother was insistent on staying. Obs 12*  *Liked the doctors approach to the interview, was very open with her questions and finding out about the child. Obs 10*  *The staff member then proceeded to administer an oral antibiotic to the child. Mum was observed to encourage the child to take medication and had proceeded to reassure her that this was the same as home. Obs 4*  *Unfortunately, some of our parents that are great advocates for children do get a label as being anxious. I worry that once they get that label then people stop listening so much, because it's easier to dismiss their concerns, because they're just anxious. Staff 2*  *I’ve seen parents completely lose it. So, I think there should be some social workers or some counsellors that should go and deal with that. I have seen - after this many times - I have seen some people being angry at staff. I’ve seen people being angry at each other. Parent 1*  *She stated that she uses her intuition and passed experience to identify children and often will ask the family to confirm her suspicions. Obs 8*  *No questions were asked around the child’s current development to flag any issues.*  *No preparation of child. Obs 4*  *His mum was very agitated. She was very protective. The moment I walked in the room, I knew there was repairing that needed to happen. Staff 2* |
| **Staff juggling multiple duties** | *Nurse instructing student how to acknowledge parent concerns, redirecting and explaining plan - “They were like ok” - nurse explains that they do a lot of de-escalation with angry parents. Obs 1*  *Observed staff members from looking very stressed and when asked how is going. stated “Thanks for asking but executive not happy”.Obs 3*  *Staff looked really busy trying to do multiple tasks and organise for patient transfers. Obs 4* |
| **Staff on reasonable adjustments** | *So, there was some confusion around that. I think in the end they gave it to him, but he vomited it up. It was unsuccessful. So, the decision was made to discharge him so that he could [go] and then bring him back through Turner [ward] as an outpatient with the option of better planning around it. Staff 2*  *It's not like staff don’t know stuff, you know, it's just something that's the gap there because if we have that understanding then we know that…behind the behavior, this is this is the way that they are responding to it just comes back to having that empathy as well by using that having that framework. Staff 3*  *Umm was thinking, you know, just based on our limited presence there and things needing to be done fast-paced with and not being able to provide much. Staff 3*  *What are find is that I* ***think*** *we're lacking in a lot of resources to assist these children and their families, just through having the lack of access…Staff 4*  *She also spoke a case she was involved with yesterday of a girl that was screaming who was waiting to be admitted to a ward but had to wait 4 hours prior to transfer. She proceeded to set a timer for her and the girl watched that timer till she moved but she stopped screaming. Obs 8*  *… it does impact your [time spent in ED], because then you've got a child in for longer. It's messy and complicated and a lot of variables at play. Staff 2* |
| **Teamwork in communication about patient** | *Also observed this type of interaction with junior to senior medical staff handover. It had a very diagnostic focus and treatment care path. Obs 8*  *Radiologist then appeared and stated that there was no request in system and child started to get upset again as they were told they would have to go back to Fast track area. Obs 5*  *… I just want to get home. So realistically, they just…. Yeah, I think when people are stressed, when they [are] rushed and they just [want to get home]….[just] wanna give the basic kind of hand over. Staff 4* |
| **Value of Child Life Therapist – supporting child, parent and staff** | *Advocating for child space, parent, their role and educating staff about their role and influencing culture change (build trust with staff, working with consultants and allow psychological safe space for staff to debrief)*  *We have had to put in a lot of work. Because of the pace and the dynamic of ED, if you're not seen you're not used. You have to be - we learnt we had to be physically there, have our feet on the ground, and we needed to be seen for them to use us. Staff 2* |

| **Post Intervention** |  |
| --- | --- |
| **Improvements noted by parents** | *She stated that staff could see straight away her child would need support. Obs 3*  *She did say that she had previously been to [x Hospital] but they did nothing and that she had better treatment and more done here. Obs 2*  *“Best experience she had so far in the hospital” Happy with experience with JMO, patient and took time, gave appropriate updates. Obs 4*  *And then she got the top 5 done, and then she (CLT) consulted with the medical team and staff.… I think it makes a difference because that never happened to me before and I think that is a brilliant thing to have in place. It was a more positive experience, and I have ever experienced before. Parent 1.*  *[Parent] did not know that the top 5 questions were available online [ED electronic health record] but agreed that they are a good description of the child. Quote from Observation 4*  *She stated that she could feel the change in staff’s interest and care she has received. Obs 3*  *She [Parent] stated that she presented in June 2023 and ‘no doubt it was so streamlined’. She felt like she was being heard and that everyone on board from the admin, nurses, doctors and even security. Quote from Observation 8.*  *I've been going to that emergency department for a very long time and she had a lot of medical problems, so I was there for a long time…..just having them [Child Life Therapist] in place was amazing. Parent 1*  *Staff were more engaging with her child with the disability and were interacting with him rather than talking over him. They were engaging him with his interests. Obs 8* |
| **Improvements noticed by staff**  .. | *It's just a really nice experience to be part of, to witness that the training that we're doing is working [or]…I would not have the motivation to continue it. I would not have [thought it would be] making a difference out there. And I think, yeah, I just think about, like, if I didn't see value in this programme, motivation to say yes, we need to implement it in our orientation program. Staff 6*  *Support for preparation noticed and staff also recognise that they have the skills to support this as well. Obs 6*  *‘So I guess after the train the trainer [program]… was good insight and for all that was just to reflect on those difficulties and to have a better approach of these patients. So definitely [it] was very eye-opening. It is just sometimes we know what to do but is just putting them altogether in a more structured way… definitely was an eye opener and definitely helps with the anxiety feeling before seeing patients’. Staff 2*  *I did come across a lot of families after the training and I did feel like because I look more confident, they were more comfortable as well in, like they were….They felt probably like…more trusting of me … when I was, like, dealing with them. After the training, I think it just gave me that boost that I needed. So I when I approached the family, I just approached him in a very calm way. Always in my mind with….like I should prioritize the needs of the child ….approaching them a bit more differently definitely helped and just made that whole interaction very smooth and actually thanked me a lot after I finished the consultation. Staff 2*  *They also mentioned that they noted that [the] Top 5 flags in Radiology and that they noted that radiology will ring to find out more about the child to assist with preparation. Obs 6*  *She also stated that she has seen a big change with the medical staff due to [local champion’s] input and finds them more accommodating to see the children and use of single rooms to assist with sensory overload. Obs 1*  *So I was involved in talking to the child. So he kind of realised that we were doing everything we could to make him comfortable. And so, he quieted down and allowed me and the surgical registrar to examine the child….So we were able to send the child home and managed the child very efficiently. And I remember at the end, the child came up say thank you … that was quite nice. Staff 1*  *Nursing staff were very proud of the outcome and pleasantly surprised at how well he tolerated the procedure. Past experience had been being held down and getting upset as described by mother. Obs 6* |
| **Increased staff enthusiasm and optimism** | *“…approaching them a bit more differently definitely helped and just made that whole interaction very smooth and actually [parents] thanked me a lot after I finished the consultation, which was very surprising to me given the fact that everyone's saying [they were] a very difficult family and they actually warned me before entering the room. So I guess I must have done something really good with that training, yeah.” Staff 2*  *“Personally, ..everything that I've heard about this programme has been amazing. Everyone's been fantastic. It works like even things like, you know, I used One voice. It really worked really well.” Staff 6*  *She also stated that she is very passionate about the project and feels that it is making a huge difference and that one day it will the standard of practice in the unit. Observation 1*  *Staff highlighted that importance of program supporting the learning that is required to fill out Top 5 and care for these children. They are now looking at how new staff can learn this as well. They were open to providing suggestion to assisting with development of Top 5 video. Observation 6*  *‘It is just a really nice experience to be part of, to witness that the training that we are doing is working …I would not have the time to make a difference out there. And I think, like if I did not see value in this program, [I would not have the motivation] to say yes, we need to implement it in an orientation program. Staff 6* |
| **Issues still persist** | *Maybe in the business of the department, maybe they didn't have enough time to spend with him and knowledge too that not, you know, realising this is not gonna work for this particular child. Staff 4*  *Doctor was overwhelmed with work and did not pick up on cues. Really was not listening to what mum was asking advise of. Obs 2*  *There may not be a single room to put them in somewhere and even giving them a slightly high category their wait time may be extended …. it's sort of nothing you can do about it. Staff 5* |
| **Types of reasonable adjustments observed** | *And just you know, how did the initial kind of this is, this is what you do is thinking about 1 voice reason for judgments and all of that. And then when I did the SIM, I was like it was amazing how much they absorbed just from the teaching that one teaching session they were already, you know implementing what they learnt from that session. Staff 6*  *Lucky in a way that our ED is very adaptable in terms of the staff, they love to welcome change and I think the mere fact that our staff have actually gone out there after the teaching, the teaching of motivated to change gone out there implemented what they learned or witnessed what they learnt, go into action, they're more likely to continue to do it. Staff 6*  *‘It was nice to see the consultant who was involved actually able to deescalate her eventually.’ Staff 2*  *Well, she got down to her level at some points as well, which was good, like getting down to the child's eye level is really good. Parent 1*  *Understanding the body language so like kind of knowing where they are on their on their escalation curve. So what are the signs that they're calm and rumbling like even words like that? Like I didn't know when I was a grad start and then, you know, once I start to grumble once I start to get to rage. Staff 6*  *Child was placed in an [open] bed space ….. and close to the desk space for doctors and nurses. Curtains were drawn to not be able to see the patient in the next bed….the other side had curtains open. Dimmed lights. Low noise level overall in the department. ObsD4*  *Along the way the child was asking questions and the nurse was interacting and speaking with the child. At one point on the journey the child was abit startled by other people and reached out and grabbed the nurses hand to continue walking even though dad was right beside him as well.Obs 12*  *Procedure then attended with no sedation and minimal restraint very successfully. Obs 6*  *I was chuffed, totally chuffed that I was able to get a back slab on him with not much needing his parents to hold him. Staff 5*  *I generally tend to ask the parents will look for them in this kind of environment and what we can do to help make that better for them, which is very difficult sometimes and is like the middle of winter is very busy here trying to do as best we can for them can be a little bit hard because some kids, they need a separate space to be. Staff 5*  *So working in a timely way, finding this out from parents and all that makes such a big difference in terms of how we can be preventing or mitigating any chances of escalations? Staff 5*  *Doing a procedure with a child whom might escalate because of the procedure, and then if when we are doing the procedure trying as much as possible to talk to the child's very calmly and one person only instead of like the whole of us, and whoever is doing the procedure to be completely silent. I guess that that really helped because I was not aware about that before the training. Staff 2*  *Personally, ..everything that I've heard about this programme has been amazing. Everyone's been fantastic. It works like even things like, you know, I used One voice. It really worked really well. Staff 6*  *Yeah, they have these nice like pictures as well that they use especially for some of our patients that are very visual, definitely help some more to understand what's gonna happen. Staff 2*  ***Tile noted to be used most occasions***  *Was stationed in ATA as the ID icon appeared in First Net and TOP 5 completed for a patient. Obs 1*  *Noted to have observed on FirstNet two children with tile activated. 1st Child was same child from the 12/1/2024 returning for ear issue that was unresolved. Had left by time I got to ED. Obs 3*  *We've had a few changes in the department where there's an [electronic] tile now which pops up and once we put, you know, if we flag the child as having intellectual disability and it there's a page that pops up which gives you the top five things that the child likes. Staff 1*  *Every time I am in [KCC] and I see that there is a child with autism. When the triage nurse goes down to see them and seemed to realise we have this puzzle piece {I ask] Did you realise this is how it works? Like you do not have to put a lot of information on because people can always add to it as time goes by. Staff 5* |
| **Environment is variable and unpredictable** | *It was like it was 10:00 o'clock in the morning or something, but it was actually quite quiet and not loud, and it was really good. Parent 1*  *This kid's crying, screaming. The nurses station is busy. It's it's loud, there's buzzing noises going off and it was a loud environment. Parent 2* |
| **Moving forwards and Program Impact**  **How can we do better?**  **Need to support parents** | *We don't have a sedation protocol. It's a by sedation, I mean not, you know putting them to sleep, but trying to have the anti anxiolytic on board sometimes you know, anticipating and giving them a little bit of midaz or a little bit of fentanyl just to quiet them down before a procedure or a or some sort of a examination assessment I think might help. So we're trying to prepare sort of a policy or. A guideline really for their staff to see. Staff 1*  *With these sort of kids, the quiet pathway seems to have worked really well for them. So trying to come up with some sort of a quiet pathway because the quiet pathways is over prescribed and I think there's a huge wait times for this. Staff 1*  *We need to think about, you know, running simulations and taking that into consideration as well. So we can best prepare our team on how to deal with this, yeah. Staff 6*  *I don't know whether that's true or not, but I said before I said you need to give the parent or the carer and the child some sort of control over their situation and by doing them, you're letting them know what you know and asking for clarity because they're the expert in their child, especially in cases like N. Parent 2*  *How I think people need to know more about how to advocate in the hospital because I was lucky enough to have that knowledge. Parent 1* |
| **Program is a game changer** | *They're motivated for change. Project has been a real game changer for us and I think it's been really helpful as the staff love this project because we still used to see a lot of, you know, kids with autism and intellectual disabilities. Staff 1* |
| **Potential for new learnings that can be shared** | *And the beauty is that we don't have much research or much information about the kids with intellectual delay and disability as well present to ED. And so this is, this is a great opportunity, a research opportunity where we can actually look at an audit, you know, look at what we have done before and how it's impacted the family.* |
