# Supplementary material for: Improving the Safety and Quality of Care for Children and Young People With Intellectual and Developmental Disability. The Motivated for Change Programme in a Paediatric Emergency Department Setting. A Mixed Methods Study
Source: Health Expect. 2026 Aug 2;29(4):e70776. doi: 10.1111/hex.70776 (PMC13429100; doi:10.1111/hex.70776)
Supplement: Supplementary file 3 — Supporting File 3 [file HEX-29-e70776-s003.pdf]

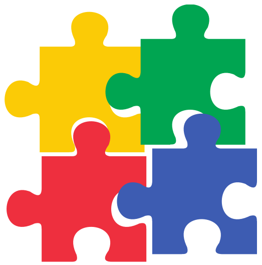

Ideas for improvement

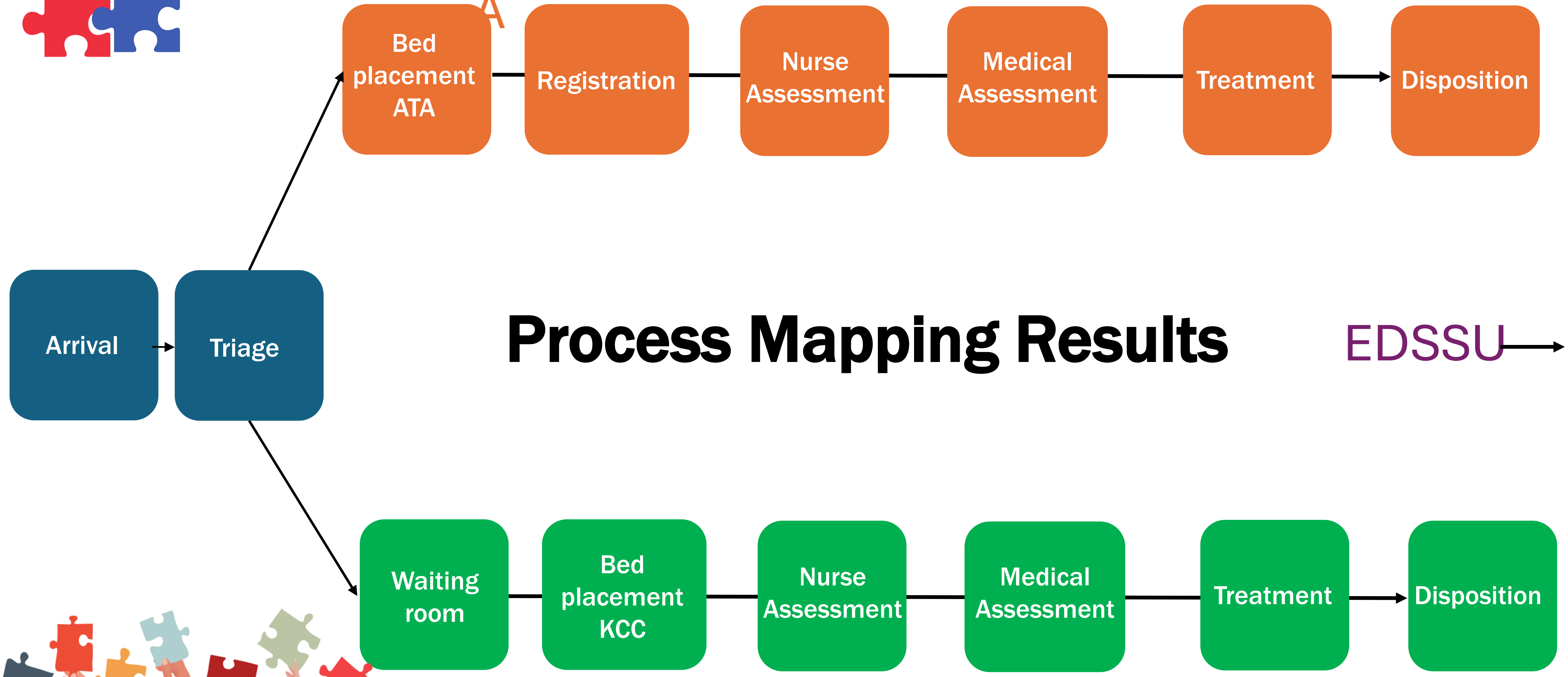

Process Mapping Results

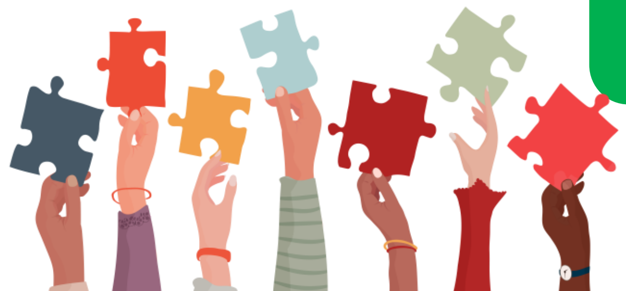

Barriers

ATA/RESUS area

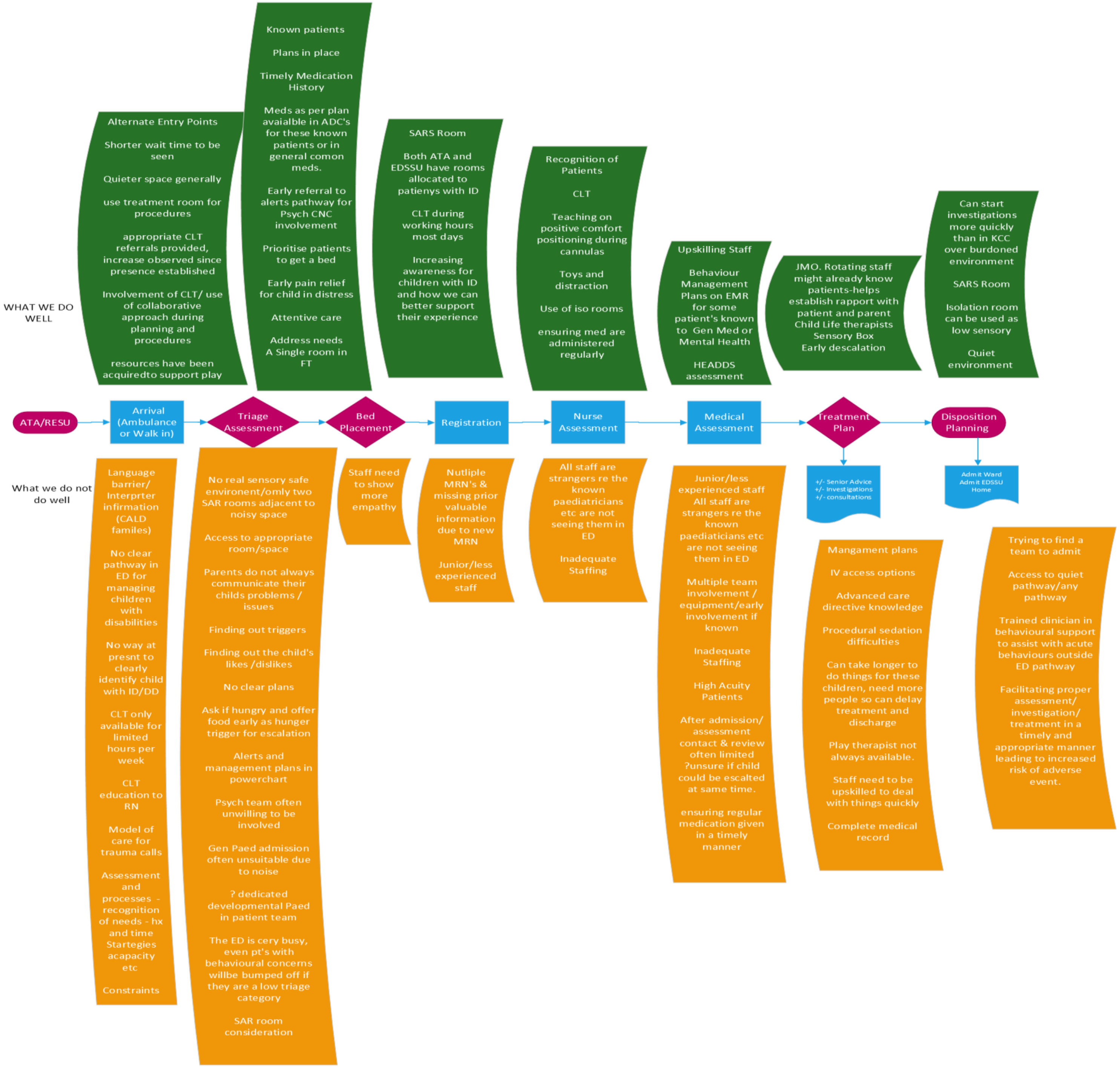

KCC Subacute

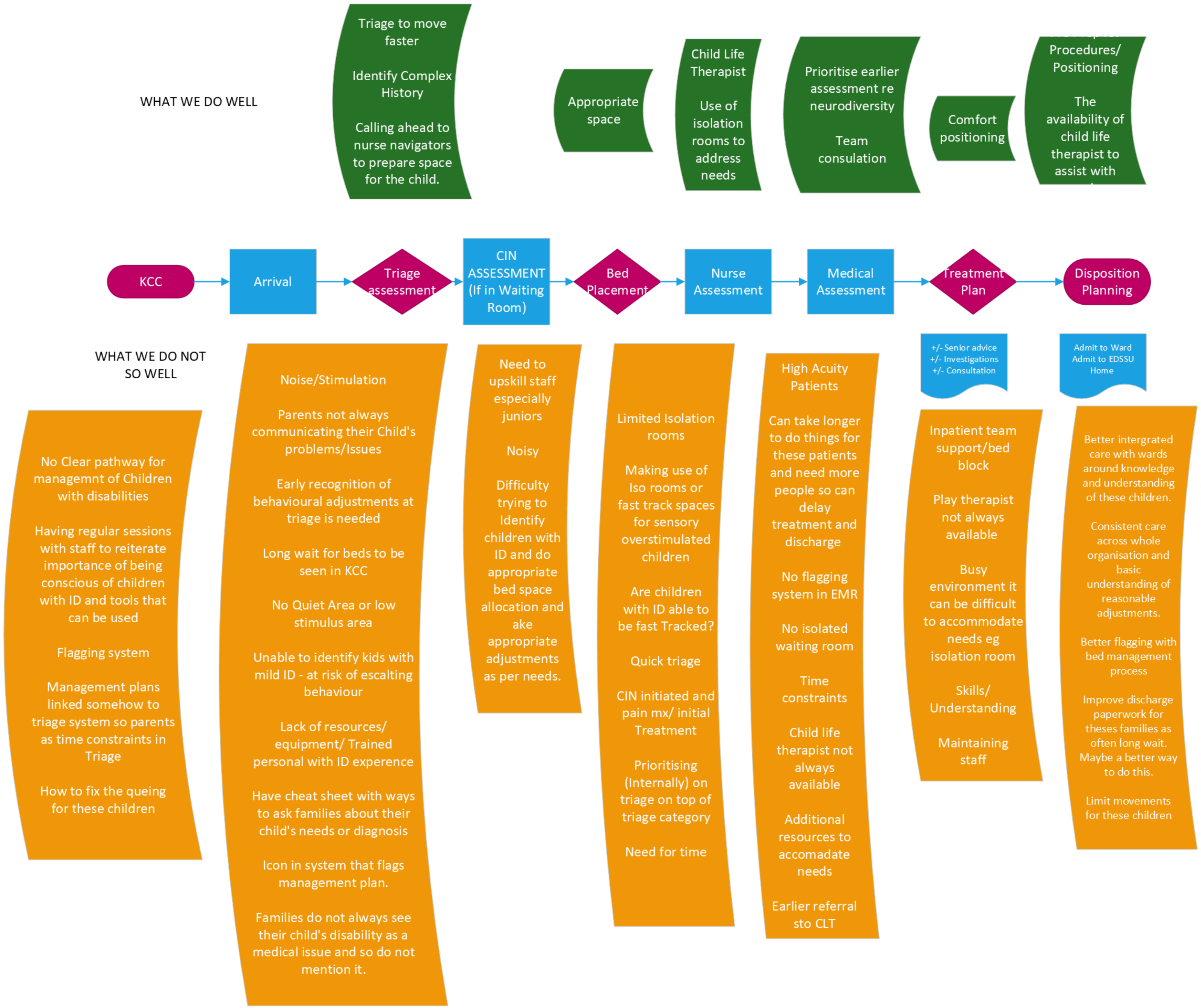

EDSSU Day Stay

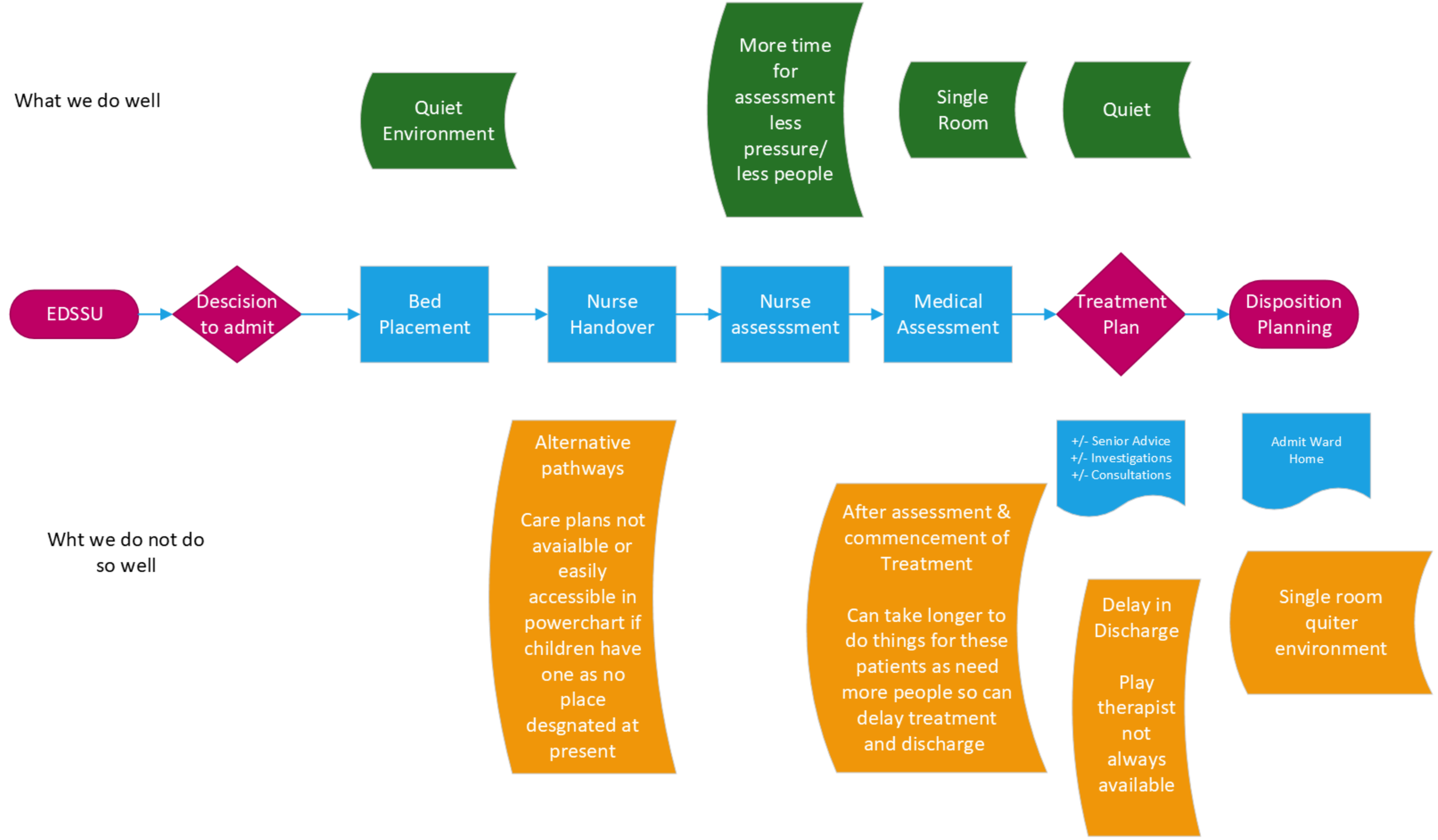

Thematic Analysis

Resources

Activities

Staffing

Environment

Processes

IT

Care Pathways

Resources

1. Visual Communication Tools
2. Sensory Toys
3. Cheat sheets | Prompt Cards
4. IPAD | TV distractors
5. Virtual Reality Goggles
6. Hidden Disability Program

Ranked in order of priority

Activities

1. Training in Adjusted Communication Methods
2. Training for JMOs and all staff
3. Child Life Therapist taking on teaching and resource development
4. Top 5 questions integration
5. Rage SIM
6. Demo of resources

Ranked in order of priority

Staffing

1. Child Life therapist to be more available
2. QR codes to visuals or videos for staff to explain procedures
3. Liaison Support officer to support child and family
4. AIN trained to provide support for nursing staff
5. Carer Advisory Group in ED

Ranked in order of priority

Environment

1. Create quiet spaces in ED (low Stimuli) with sensory toys
2. Availability of single rooms how it is used throughout the say
3. Designated places for aids e.g. visual aids, social stories, headphones and sensory toys
4. Separate waiting area with sensory toys
5. Separate triage bay, less stimulating and quieter

Ranked in order of priority

Processes

1. Early investigations / early conversations with parents
2. Triage TOP5 or management plan - ensure documents handed over
3. Early involvement in consults and triage
4. Pre-planning in the context of ED
5. Information for journey boards
6. Consider high triage category and place in quiet area
7. Sedation for these children
8. Colour coding systems to assist with flagging and paths
9. Lanyard or striker (hidden disability program)
10. Introduce strategies to handovers to wards
11. Ward nurse to come to ED to facilitate transfer of care

Ranked in order of priority

IT

1. PowerChart prompts care plans | TOP 5
2. Icon on Firstnet board to indicate child has ID or DD
3. Hospital passport to become part of EMR
4. Documentation template to be built in EMR

Ranked in order of priority

Care pathways

1. **Specific pathways for de-escalation**
2. **Procedural analgesia pathways to bypass long waiting times for prescriptions, priorities transfers to wait areas**
3. **Guidelines in the use of sedation (alternative to nitrous)**
4. **Improving discharge process to prevent extended waiting**
5. **Handover guide with ward to include TOP5 and plan. Look to getting ward staff to meet child prior to transfer**
6. **Communication pathways to alert teams or areas of childs arrival and build pathway to transition**
7. **Traffic codes for identifying emotions in children with ID**
8. **Guidelines of bed allocation to best accommodate children with intellectual disability**
9. **Posters or flats to remind about info sharing around child and transition to radiology**

Ranked in order of priority
