# Supplementary material for: Improving the Safety and Quality of Care for Children and Young People With Intellectual and Developmental Disability. The Motivated for Change Programme in a Paediatric Emergency Department Setting. A Mixed Methods Study
Source: Health Expect. 2026 Aug 2;29(4):e70776. doi: 10.1111/hex.70776 (PMC13429100; doi:10.1111/hex.70776)
Supplement: Supplementary file 4 — Supporting File 4 [file HEX-29-e70776-s004.docx]

# Summary of Quality Improvement Cycles M4CED

| **QI Cycle** | **Aim** | **Plan** | **Do** | **Study** | **Act** |
| --- | --- | --- | --- | --- | --- |
| 1. Enhancing Accessibility of Educational Materials | Improve access to pre-learning materials | Identified login and desktop-only barriers | Removed login restrictions; enabled mobile access; added QR code posters | Improved uptake; learners better prepared | Standardised QR and mobile-friendly formats for all sessions |
| 2. Optimising Teaching Format for ED Workflow | Increase attendance and engagement during clinical shifts | Long sessions limited participation | Condensed to 40-minute interactive sessions | Higher engagement and attendance | Adopted shorter, opportunistic sessions alongside protected teaching |
| 3. Contextualising Content to ED Setting | Improve relevance of education | Content was too generic | Revised materials with ED-specific literature and case examples | Greater relevance; more practice change suggestions and conversations | ED-contextualised content integrated across all resources |
| 4. Improving Simulation Fidelity and Participation | Enhance simulation authenticity and staff engagement | Needed authentic scenarios for behaviours of concern | Introduced pre-briefs, simulation aids, and refined structured debriefs | Higher engagement and spontaneous use of strategies | Standardised realistic aids and structure in simulations |
| 5. Portable Learning Aids – Staff Lanyards (“PRACTICE”) | Provide quick-reference clinical tools | Staff needed learning reminders on shift | Created and distributed summary lanyards | Lanyards used effectively and for peer teaching | Standardised and embedded in post-training resources |
| 6. Backside Lanyard Enhancements | Maximise lanyard utility | Suggestions to expand with practical tools | Added Top 5 Questions, Behavioural Curve, One Voice tool | Increased clinical use | Included in staff orientation packs |
| 7. Online Prompts for “All About Me – Top 5” Forms | Improve form completion and meaningfulness | Staff unsure how to fill open-ended fields | Added PRACTICE-based prompt examples elucidated in lanyards production. | Higher completion rates; more meaningful content | Standardised prompts and added link provided to detailed resources and instruction. |
| 8. EMR Flag for Reasonable Adjustments | Improve recognition of patients needing adjustments | Flower icon unclear to staff | Trialled icons; selected puzzle piece | Better recognition and response | Implemented puzzle icon across EMR system hospital-wide |
| 9. Improving Access to Social Stories | Standardise and digitise procedural support tools | Paper-based stories inconsistently used and difficult to access | Digitized stories; added QR code access across ED | Increased use | Social stories digital and paper versions available on all clinical areas in ED as well as on ED intranet for printable versions. |
| 10. Access to Procedural Sedation Resources for Children with ID/DD | Improve staff confidence in ID/DD sedation support | Staff lacked consolidated resources | Updated education and policy; added intranet section | Greater confidence; proactive non-pharmacological strategies | Embedded content into policies procedural sedation teaching, and ED intranet |
| 11. Enhancing Simulation for Severe Behavioural Escalation | Improve training for high-risk ID/DD presentations | Needed authentic escalation scenarios | Co-designed simulations with families and specialists | Identified need for lived experience integration | Further scenario iterations to include lived experience voice from person with ID/DD |
